# Gene expression analysis method integration and co-expression module detection applied to rare glucide metabolism disorders using ExpHunterSuite

**DOI:** 10.1038/s41598-021-94343-w

**Published:** 2021-07-23

**Authors:** Fernando M. Jabato, José Córdoba-Caballero, Elena Rojano, Carlos Romá-Mateo, Pascual Sanz, Belén Pérez, Diana Gallego, Pedro Seoane, Juan A. G. Ranea, James R. Perkins

**Affiliations:** 1 Department of Molecular Biology and Biochemistry, University of Málaga, Bulevar Louis Pasteur 31, 29010 Málaga, Spain; 2 Centro de Investigación Biomédica en Red de Enfermedades Raras (CIBERER), Valencia, Madrid, Málaga, Spain; 3 Departamento de Fisiología, Facultad de Medicina y Odontología, Universidad de Valencia-INCLIVA, Valencia, Spain; 4 Consejo Superior de Investigaciones Científicas, Instituto de Biomedicina de Valencia, Jaime Roig 11, 46010 Valencia, Spain; 5 Centro de Diagnóstico de Enfermedades Moleculares, Centro de Biología Molecular-SO UAM-CSIC, Universidad Autónoma de Madrid, Campus de Cantoblanco, Madrid, Spain; 6 Instituto de Investigación Sanitaria IdiPaZ, Madrid, Spain; 7 Institute of Biomedical Research in Málaga (IBIMA), Calle Dr. Miguel Díaz Recio, 28, 29010 Málaga, Spain

**Keywords:** Computational biology and bioinformatics, Cellular signalling networks, Computational models, Software, Statistical methods, Transcriptomics, Gene expression profiling, Gene expression analysis, Mechanisms of disease, Metabolic disorders, Neurological disorders

## Abstract

High-throughput gene expression analysis is widely used. However, analysis is not straightforward. Multiple approaches should be applied and methods to combine their results implemented and investigated. We present methodology for the comprehensive analysis of expression data, including co-expression module detection and result integration via data-fusion, threshold based methods, and a Naïve Bayes classifier trained on simulated data. Application to rare-disease model datasets confirms existing knowledge related to immune cell infiltration and suggest novel hypotheses including the role of calcium channels. Application to simulated and spike-in experiments shows that combining multiple methods using consensus and classifiers leads to optimal results. ExpHunter Suite is implemented as an R/Bioconductor package available from https://bioconductor.org/packages/ExpHunterSuite. It can be applied to model and non-model organisms and can be run modularly in R; it can also be run from the command line, allowing scalability with large datasets. Code and reports for the studies are available from https://github.com/fmjabato/ExpHunterSuiteExamples.

## Introduction

RNA sequencing (RNA-seq) is widely used across molecular biology and biomedicine, including rare disease research^1^. However, different experimental designs, sequencing protocols and technologies mean that the properties of the output data can vary greatly. A single analysis package is rarely sufficient to ensure robust analysis^2^.

**Figure 1:**
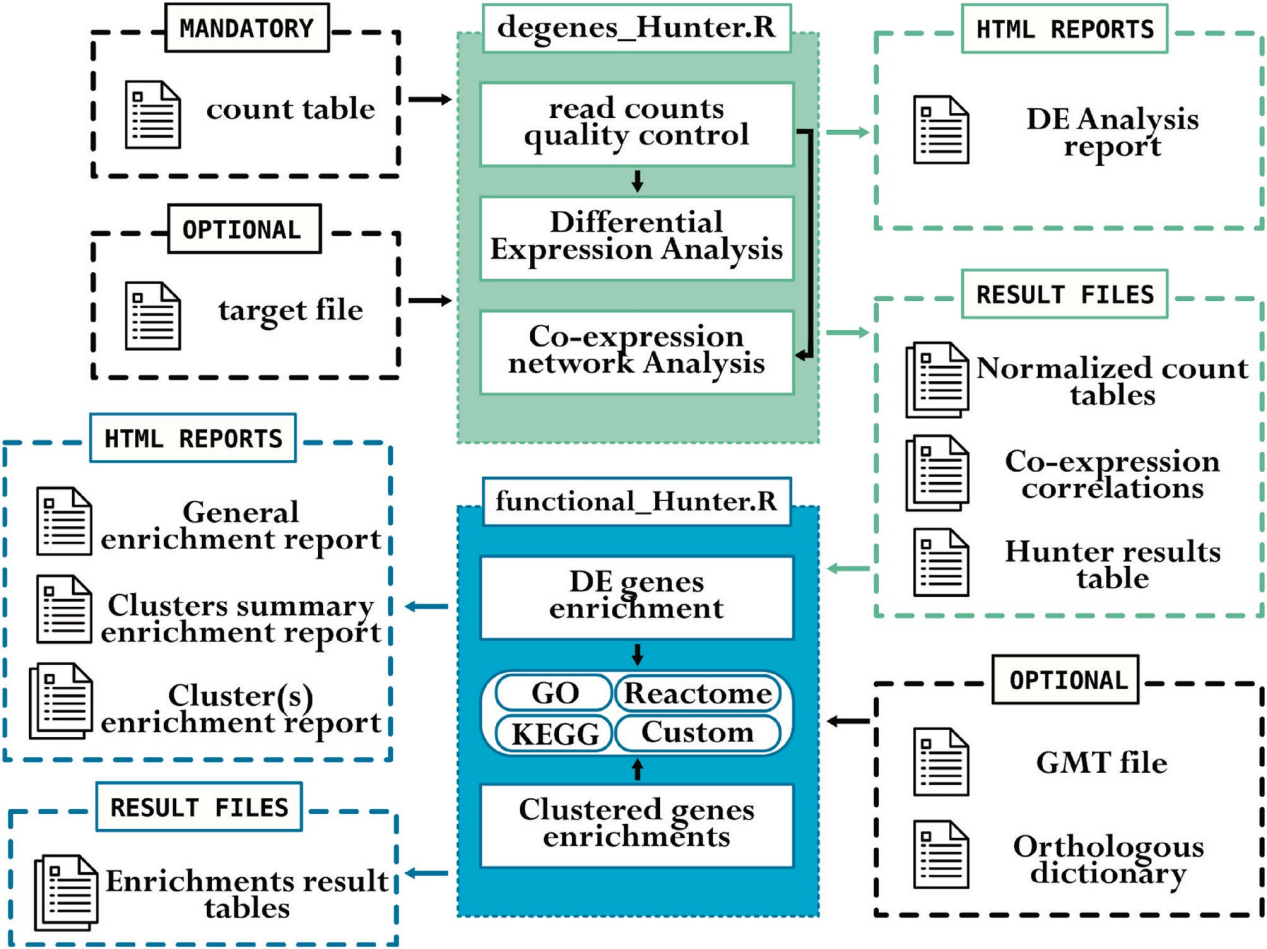
Overview of the workflows implemented in ExpHunter Suite and their input/output. The green box represents the DEgenes Hunter module related to differential expression analysis; the blue box represents the functional Hunter module related to functional enrichment analysis. Boxes with dashed borders represent input and output files, including html reports.

Various workflows exist for initial RNA-seq data analysis to produce a table of counts, which serves as input for downstream processes such as differential expression (DE) analysis^3,4^. DE methods are based on different assumptions and analysis procedures, making it impossible to know the most appropriate method for a given dataset^5^. This has led to the appearance of workflows that include multiple differentially expressed gene (DEG) detection packages^6^. Previous studies have looked at combining results of multiple DEG methods to improve DEG detection, using consensus and ranking based strategies as well as p-value integration^7–9^. However, to our knowledge no previous work has applied machine learning based classification methods to this problem. Detected DEGs serve as input for functional enrichment analysis, in which lists of genes are converted into biological knowledge^10^. Although protocols suggest using functional enrichment in addition to DE analysis, few packages implement both^3^. Fewer still combine multiple annotation databases and custom term sets. Co-expression analysis, which searches for groups of co-expressed genes (CEGs) that correlate with phenotypic data^11^, is also often overlooked in RNA-seq data analysis, despite its potential for better understanding molecular processes and disease^12^. It can be used as an alternative to DEG detection, or as a complementary analysis technique. Here we present a comprehensive methodology for the analysis of transcriptomic data. We provide a collection of tools, the ExpHunter Suite, implemented as an R/Bioconductor package including auxiliary scripts for assessing performance and simulating RNA-seq data. It incorporates the DEgenes Hunter pipeline^13^, in addition to co-expression analysis, multiple reports related to quality control and result interpretation, and provide ways to compare and combine results. It can be used with and without reference genomes and has been applied to a range of species^14–19^, with annotation being provided through orthologous translation to perform functional analysis of non-model organisms, as demonstrated in previous work involving our group^20^. It is also possible to specify multifactorial experimental designs and control for additional factors. An overview of the methodology is given in Fig. 1.

We apply it to simulated and spike-in and real datasets, showing that some widely-used expression analysis methods can behave quite differently depending on the properties of the data, potentially over-predicting or estimating inaccurate values. We provide novel methodology to combine results, including a Naïve Bayes classifier approach, that lead to robust DEG detection. The real datasets are derived from two experiments modelling rare diseases related to carbohydrate metabolism. We use the package to confirm existing knowledge related to immune cell-infiltration in Lafora disease and calcium channel involvement in PMM2-CDG, and suggest related genes for further study. Through co-expression analysis, we find examples of divergent expression patterns between mRNA transcript and protein levels for the same gene, detect genes related to the extracellular matrix with a potential role in PMM2-CDG and modules of genes including triggers of NK-$\kappa$B and MAPK processes in Lafora disease. These finding show the capability of our methodology to detect novel genes and functions for further study.

## Results

### Performance of differentially expressed gene detection methods using simulated datasets

DE analysis was performed on a range of simulated expression datasets, to evaluate how different properties of the dataset can affect the performance of DEG detection and combination methods. Multiple expression datasets were simulated based on the two rare disease RNA-seq experiments described in this article and an *A. thaliana* dataset from the R package TCC^21^.

**Figure 2:**
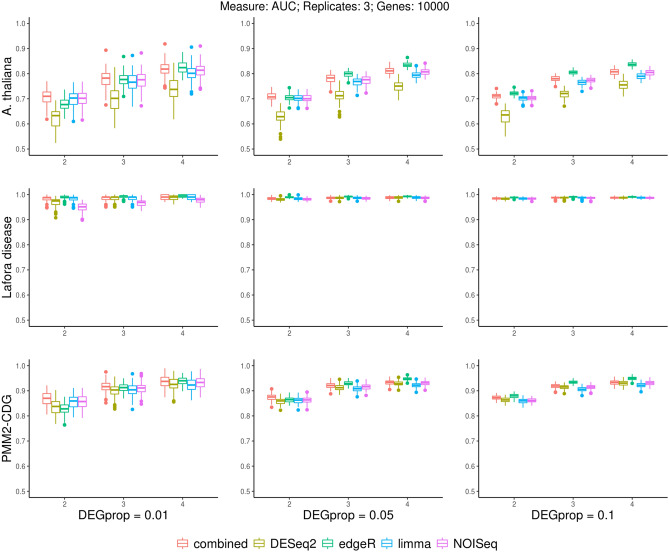
Boxplots showing area under the curve (AUC) for different DEG detection methods applied to a range of simulated datasets. The datasets shown include three replicates and 10,000 genes. Plots are grouped by experiment from which the simulated dataset was derived (rows) and proportion of simulated DEGs (columns). Within each plot different values of simulated log2 fold change values for the DEGs are shown (x-axis). Boxplot colours represent different DEG detection methods.

For the 108 combinations of parameters described in Supplementary Methods, Table 1, 100 datasets were simulated per experiment, leading to 24,300 datasets. We compared the DEGs detected by the different methods to the simulated DEGs (Fig. 2). This figure also shows the performance obtained when using combined-FDR values, which are based on the results of the different methods, as described in “Materials and methods” section. We focus on the results for 10,000 genes and three replicates, similar trends were found with other parameter combinations (Supplementary Report 1).

All packages show similar performances in most situations, with no single method performing the best in all scenarios (Fig. 2). Importantly, the combined-FDR method tends to perform well in general. Performance improves when the simulated logFC for the DEGs is greater. Notably, increasing the proportion of DEGs does not lead to better performance, but less variation in performance across replicates. The properties of the experiment used to generate the simulated data is also a key factor, with all methods performing better for the simulations based on the Lafora disease experiment. Previous studies have shown the influence of the underlying dataset in expression data-analysis, such as the MAQC-II study, in which multiple predictive models were built based on gene expression data to classify a sample with respect to disease-related endpoints^22^.

**Figure 3:**
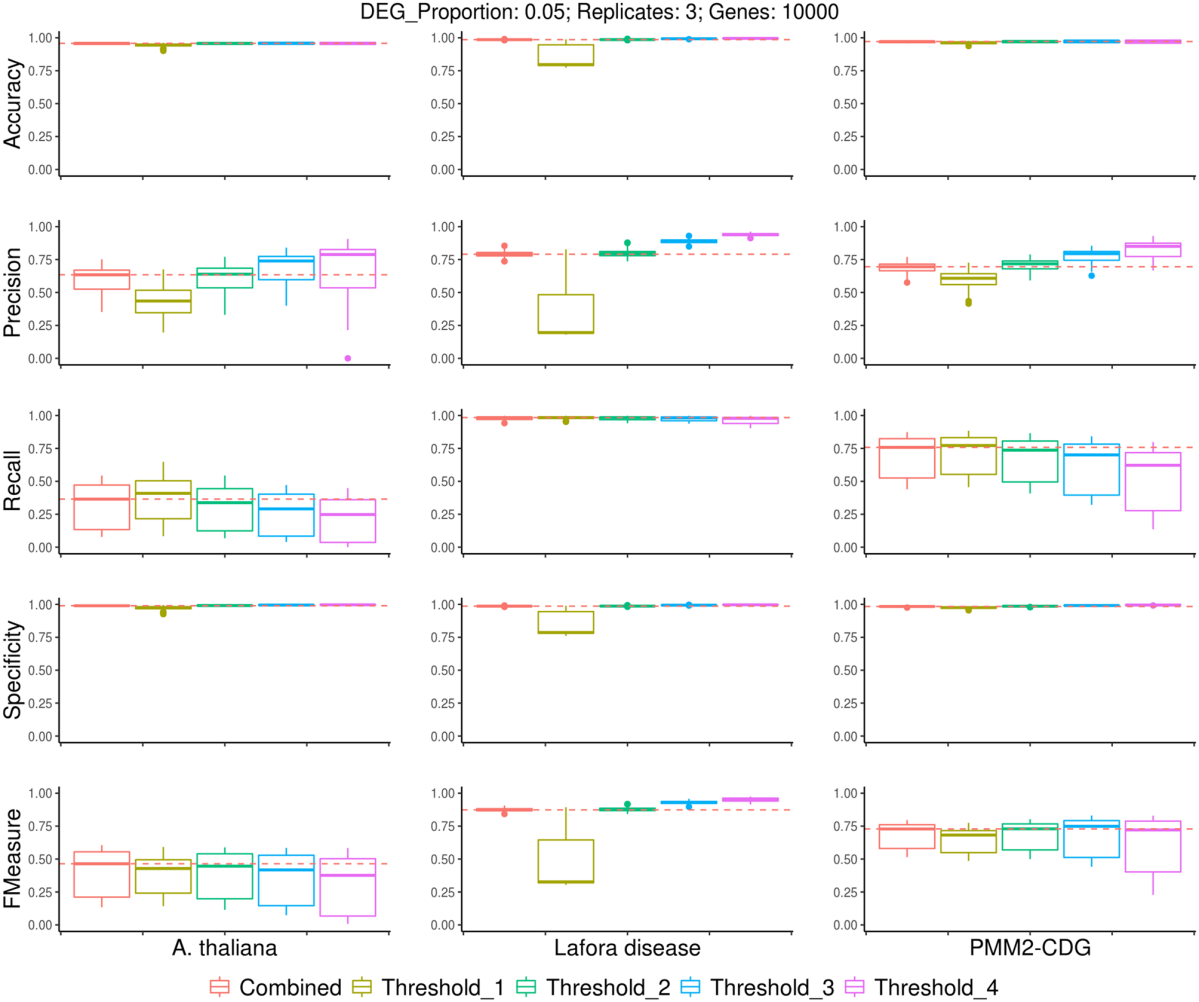
Boxplots showing performance metrics for different combination methods across a range of simulated experiments. The simulated datasets shown included three replicates, 10,000 genes and a simulated DEG proportion of 0.05. Plots are grouped by metric (rows) and experiment (columns). Boxplot colours represent the different combination methods used. The broken horizontal red line represents the median value for the combined-score system. *Recall* sensitivity, *precision* positive predictive value (PPV). Threshold refers to the minimum-vote threshold used for combining the results of different methods.

Our methodology allows the use of a consensus based threshold to ensure robust results, based on the number of methods detecting a given gene as DE. This minimum-vote threshold is further described in “Materials and methods” section. We applied this to the simulated datasets (Fig. 3), investigating different minimum-vote thresholds and comparing results with the combined-FDR. Using a one package threshold leads to reduced performance across most measures except recall. Increasing the threshold leads to increased precision at the cost of recall. The combined-FDR method performs comparatively well across all measures, obtaining similar results to a minimum-vote threshold of 2.

Better results are observed for the Lafora disease experiment derived simulations across most measures, with the exception of the vote system with a minimum-vote threshold of 1. As with the individual package analysis, this is followed by the PMM2-CDG and *A. thaliana* experiments. In terms of F-measure, defined as the harmonic mean of precision and recall, also known as F1-score,, this gradually increased with larger minimum-vote thresholds for Lafora disease, whilst the opposite was true for *A. thaliana*. This shows the importance of taking the properties of the dataset into account in DE analysis. It also suggests that there is no single best strategy for all experiments.

### Spike-in transcript detection

We applied the methodology to a publicly available RNA-seq dataset derived from samples to which known quantities of endogenous RNA had been added. Three groups of mice received different mixes corresponding to different quantities of transcripts of known genes; a fourth group of samples received no mix. For DEG detection, we focused on the comparisons between samples receiving mixes, as these represented more subtle changes in gene expression than those involving the no mix receiving samples.

**Figure 4:**
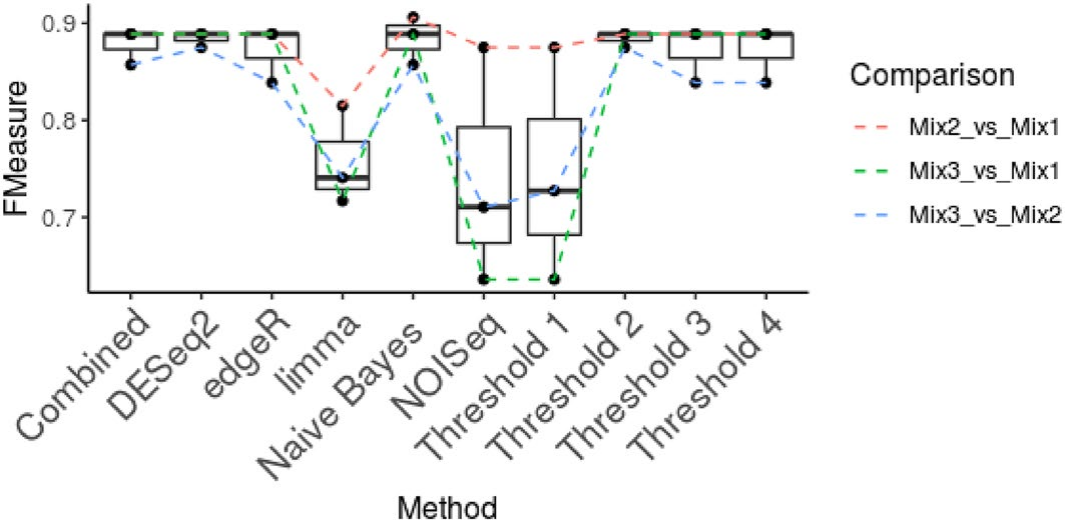
Performance of the different DEG detection methods and vote system to detect DEGs. Boxplots showing the distributions of F-measure values for each method and combination approach. Individual data points are shown and connected according to the comparison between spike-ins for which they were calculated. Combined refers to the combined-FDR results. Threshold refers to the minimum-vote threshold for combining method results.

We calculated F-measure in order to compare the performance of the DEG detection methods and combination strategies (Fig. 4). Full details are shown in Supplementary Report 2. In terms of the individual methods, F-measure was highest for DESeq2. As with the simulated datasets, combined-FDR tends to perform well in most situations. Similar trends were found for AUC, except that limma performed better than edgeR (Supplementary Results—Methods Comparison Fig. 1A). Average performance is summarised in Table 1.

For the vote system, a threshold of only one DEG detection method leads to the highest recall, as expected (Table 1). This comes at the cost of precision, which is lower than for all other measures with the exception of NOISeq. Once the threshold is raised to 2, precision increases greatly, without causing much reduction in recall. Notably, precision does not increase further with the use of stricter thresholds. The Naïve Bayes approach achieves top performance in terms of F-measure, comparable to the consensus approach with a cut-off of 2 and DESeq2. For this analysis, the Naïve Bayes model was trained using simulated data-sets based on the characteristics of the Lafora dataset.

**Table 1:** Average performance of the different methods across multiple metrics.

	FP	FN	Precision	Recall	F-measure	AUC
Combined	4.33	3.33	0.86	0.89	**0.88**	**0.98**
DESeq2	4.33	**3.00**	0.87	**0.90**	**0.88**	**0.98**
edgeR	4.33	3.67	0.86	0.88	0.87	0.96
limma	**2.33**	10.67	**0.90**	0.66	0.76	**0.98**
NOISeq	17.33	3.33	0.65	0.89	0.74	0.96
Threshold 1	17.33	**3.00**	0.65	**0.90**	0.75	–
Threshold 2	4.33	**3.00**	0.87	**0.90**	**0.88**	–
Threshold 3	4.33	3.67	0.86	0.88	0.87	–
Threshold 4	4.33	3.67	0.86	0.88	0.87	–
Naïve Bayes	4.33	**3.00**	0.87	**0.90**	**0.88**	–

*FP* false positives, *FN* false negatives, *AUC* area under the curve, *recall* sensitivity, *precision* positive predictive value (PPV). AUC is not shown for the threshold and Naïve Bayes methods as only single values of sensitivity and specificity could be produced with these methods. Numbers in bold represente the highest values for each metric.

In terms of the estimated logFC, all methods showed similar correlation with the known values (Table 2). Here we included samples with no mix added, as the logFC values should theoretically be infinite in this case, however the different DEG detection methods deal with this problem in distinct ways. To allow for comparison with the real values, transcripts in the no mix samples were arbitrarily ascribed values of 1 attomoles/microlitre. Full details are shown in Supplementary Report 3. Correlation between estimated and known logFC values for these comparisons was worse than for the comparisons not involving the no mix sample (Table 2). Of note, as shown in Supplementary Results—Methods Comparison Fig. 1, panels B and C, we see that many non-significant genes in limma are indeed changing in expression by a large amount, whilst conversely using edgeR, almost all genes that have a relatively high logFC are significant, in line with the Venn diagram (Supplementary Results—Methods Comparison Fig. 2), illustrating the importance of running multiple packages and investigating the results.

**Table 2:** Correlation between the estimated log2 fold change values from the differentially expressed gene detection methods and the known log2 fold change values for all spike-in sample comparisons, followed by the mean and standard deviation (sd) for each method. M1–3 refer to the samples with the corresponding mixes added; N refers to the sample to which no mix was added.

	M2/M1	M3/M1	N/M1	M3/M2	N/M2	N/M3	Mean	sd
DESeq2	0.96	**0.98**	0.78	**0.98**	0.84	0.88	0.90	0.08
edgeR	0.96	**0.98**	0.79	**0.98**	0.85	0.88	0.91	0.08
limma	**0.97**	**0.98**	**0.83**	**0.98**	**0.87**	**0.89**	**0.92**	**0.07**
NOISeq	0.96	**0.98**	0.82	**0.98**	**0.87**	**0.89**	**0.92**	**0.07**
mean	0.96	**0.98**	0.81	**0.98**	0.86	**0.89**	0.91	**0.07**

The comparisons are shown such that n/m refers to comparison between n and m. For example, M2/M1 refers to the comparison between mix 2 and mix 1. Numbers in bold represente the highest values for each metric. Numbers in bold represente the highest values for each comparison.

### Differences in log2 fold change estimation between methods using real datasets

**Figure 5:**
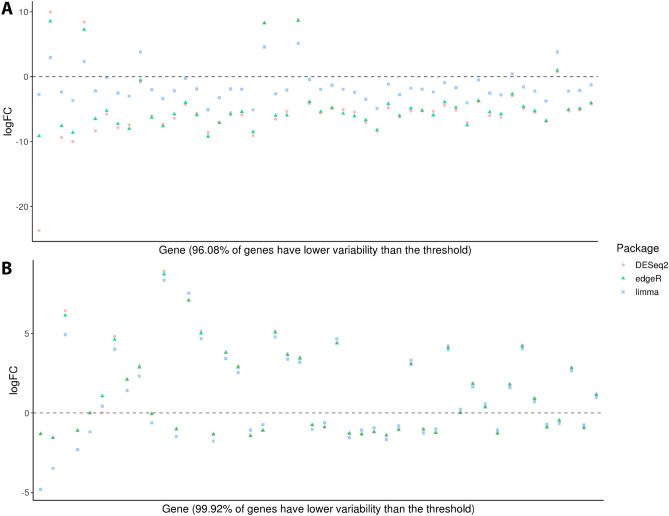
Log2 fold change values according to the different DEG detection methods for a subset of genes from the (**A**) PMM2-CDG and (**B**) Lafora disease datasets. Genes chosen based on the variance of estimated log2 fold change values across all three methods ($\ge 0.01$). Genes ordered along the x-axis by decreasing variance. The Lafora disease dataset showed relatively few genes with significant variance in terms of logFC.

When applied to the rare disease datasets, the DEG detection methods showed remarkable differences to each other in terms of estimated logFC in a number of situations. This is illustrated in Fig. 5. Notably, the PMM2-CDG dataset, shows a much larger number of genes with significant variance, with one gene obtaining logFC values of − 23.5, − 8.8 and − 2.6 for DESeq2, edgeR and limma respectively. In general, limma estimated lower values. This graphic is included in the output reports as a way of identifying potential outlier genes and, in more extreme cases, problems in a dataset.

### Real dataset analysis

#### PMM2-CDG

The methodology was applied to the PMM2-CDG dataset. Full details of the analysis are given in Supplementary Results—Case Studies and the ExpHunter Suite generated report in Supplementary Report 4. They highlight the importance of running exploratory plots such as PCA and comparing the results of the different gene detection methods. Interestingly, of the three DEG detection methods run on this dataset, 1081 DEGs were detected by at least one of them, but only 345 were detected by all three, with DESeq2 detecting many more DEGs. Given the high variability between the different methods in terms of different numbers of DEGs detected, we used a minimum vote threshold of three to determine prevalent genes for downstream functional analysis.

**Figure 6:**
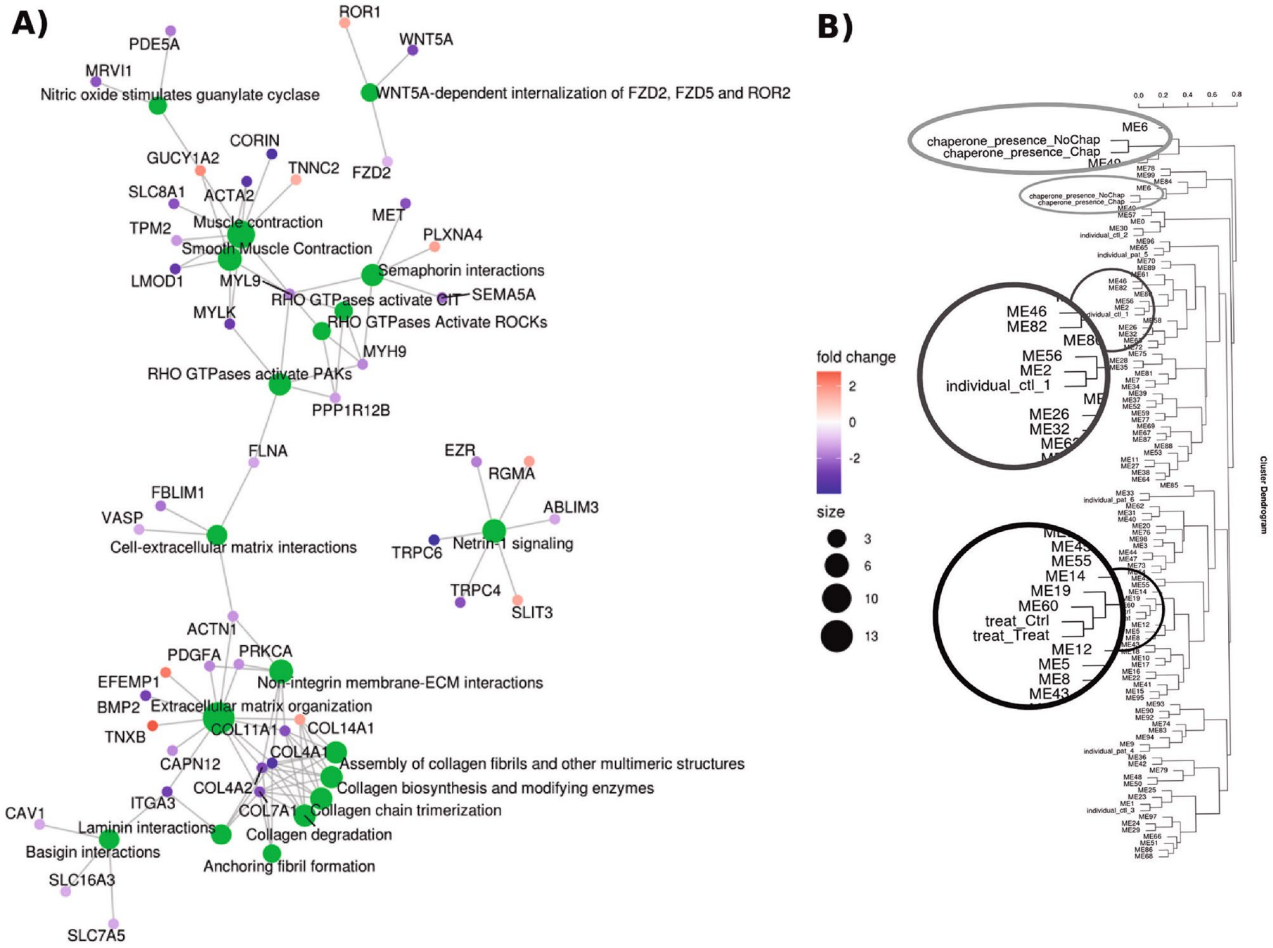
Example plots taken from the PMM2-CDG study report produced by ExpHunter Suite. (**A**) functional enrichment plot for GO Biological Processes showing overrepresented categories and genes amongst DEGs. (**B**) Dendrogram based on correlation between the co-expression modules and the categorical vectors, calculated as described in “Materials and methods” section.

The functional enrichment results point to the alteration of the formation and composition of basement membranes in the disease, due in part to extracellular matrix and collagen related processes, including impaired collagen IV network formation, associated with PMM2-CDG related symptoms, intra-cerebral haemorrhages and stroke like episodes^23,24^. Figure 6A shows these processes, linked by genes shown to be significantly DE in the experiment. These results are reinforced by the cluster analysis, which finds a module of genes highly correlated with the treated samples (Fig. 6B), which shows enrichment for functions related to cell secretion, in line with the involvement of the extracellular matrix found in the more general enrichment studies. Notably, through network analysis we find *CACNA1H*, highly underexpressed in the disease samples, which encodes a calcium channel involved in various developmental disorders^25^. Other calcium channels, such Cav2.1, have been reported to be involved in PMM2-CDG cerebellar syndrome and proposed as a therapeutic target^26^.

#### Lafora disease

The utility of our package is further emphasized by the results of its application to the Lafora disease dataset, full details of which are given in Supplementary Results—Case Studies. The ExpHunter Suite generated report is shown in Supplementary Report 5. Exploratory analysis showed that disease and control animals formed separate groups, with further subdivision between the different mutants. In contrast to the PMM2-CDG analysis, DESeq2 did not detect any genes that were not be found by the other methods.

**Figure 7:**
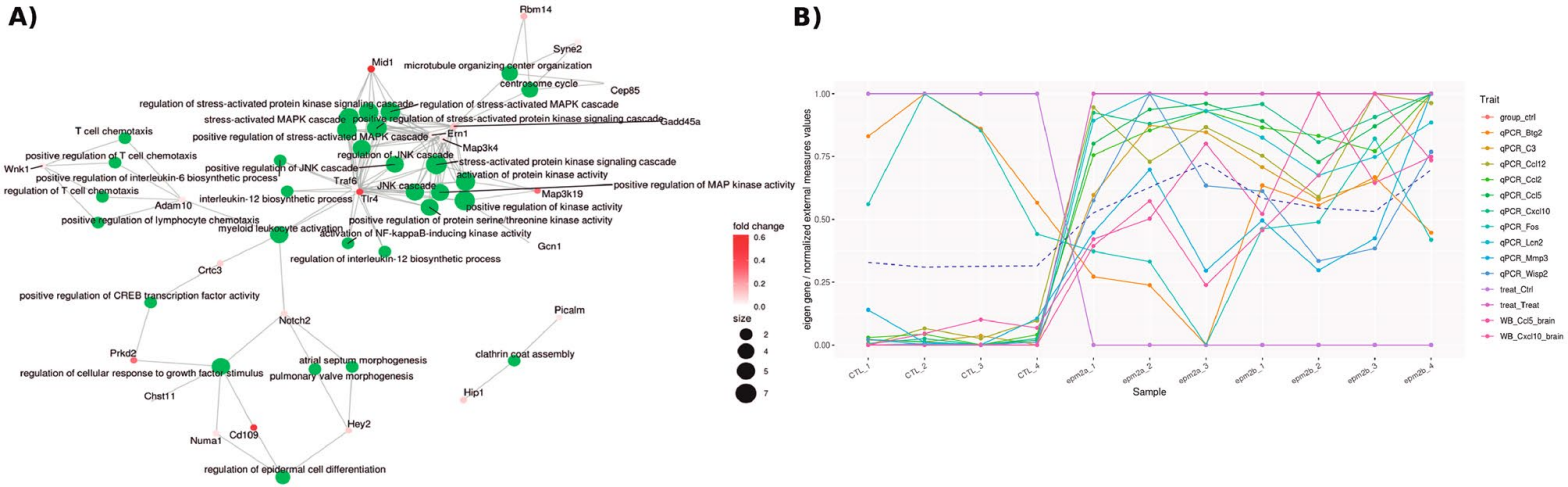
Example plots taken from the Lafora disease study report. (**A**) Functional enrichment plot for GO Biological Processes overrepresented among genes in co-expression module 23. (**B**) Eigen-gene values for module 1, which represent averaged expression values for the genes within the module, as described in “Materials and methods” section, alongside external measure values for all significantly correlated categorical and continuous vectors significantly correlated with the eigen-values (p-value < 0.05), calculated as described in “Materials and methods” section.

Results of the enrichment analysis applied to all DEGs confirmed previous findings related to microglia-astrocyte cross talk in neurodegeneration^27^. Further analysis of the clusters highly correlated with the mutant animals found similar results, including immune system and inflammatory processes known to be important in the disorder and suggesting potential targets for further study and helping better understand its underpinning mechanisms (Fig. 7A)^28^ Supplementary Report 6 shows the ExpHunter Suite generated report for one of these clusters.

This analysis also demonstrates the utility of the clustering for detecting potential outliers, identifying a module of genes that correlates strongly with a single sample. It also identifiers transcripts and proteins for the same gene showing correlation with distinct co-expression modules (Fig. 7B), potentially indicative of post-translational modifications.

## Discussion

It is clear that there is no single method for DEG detection that can be recommended as a one size fits all solution. Amongst the spike-in and simulated dataset analyses, for which ground-truth DEG expression values can be calculated, the top performing method varies between datasets. This becomes even more marked for the real datasets. For example, the Lafora disease experiment, showed DESeq2 was the most conservative method, not predicting any additional genes that were not found by at least one other method (Supplementary Results—Case Studies Fig. 5B). Conversely, for the PMM2-CDG experiment DESeq2 was by far the least conservative, predicting hundreds of DEGs not found by either of the other methods. In general, NOISeq and DESeq2 showed high recall, whereas limma showed higher precision.

Given these finding, our methodology includes various methods for DEG detection. Our intention here was not to perform a comprehensive comparison of DEG detection methods under multiple scenarios and for different implementations; these already exist^29–31^. Instead we suggest a somewhat agnostic approach, making use of the multiple reports produced by our package. Moreover, we would suggest the use of a minimum vote threshold cut-off where possible. As we have shown, increasing this threshold leads to improved precision at the cost of recall. Although for the spike-in data the optimum threshold was 2 in terms of F-measure, this may differ for other organisms, tissues and experimental designs. As such, we are hesitant to extrapolate these findings to a more general rule, especially given the effects of differences in dataset properties as described above. Moreover, it should be noted that another study has recommended much higher thresholds, although this was based on the analysis of a single human dataset^6^.

The Naïve Bayes classifier presented here performed as well as the best performing single and combination methods on the spike-in dataset. Training the classifier using the simulated experiments based on the Lafora disease dataset, we have allowed it to learn from a wide range of potential scenarios in terms of numbers of differential expressed genes, magnitude of fold change and more. We have focused on Naïve Bayes here because this model can be trained in a matter of seconds; further work could look at more elaborate classifiers. This work opens up a new window into the use of machine learning.

In terms of fold change estimation, methods could vary by orders of magnitude for some genes. By identifying such discrepancies, our methodology can identify outlying genes and samples. Fold change is an important criterion when selecting genes for confirmation using techniques such as qPCR, as such its incorrect estimation could lead to poor target selection. This also has important implications for downstream analyses that use logFC, such as the popular GSEA method^32^.

A key component of the ExpHunter Suite, and a step overlooked in many protocols that deal with RNA-seq data, is the implementation of WGCNA to detect CEG modules, and their relationship with external variables pertaining to the samples^11^. Other software exists to implement such methods^33^, however they are dedicated to this purpose only, requiring the user to seek other methods and protocols.

Here we have shown, using two datasets with distinct designs and from different organisms, how co-expression analysis can be used to add an important extra facet to RNA-analysis. It allows us to confirm existing hypotheses and speculate novel ones. We also underline the importance of including functional enrichment when analysing the modules, allowing us to find things that were not necessarily found with DE analysis. For example, for Lafora disease, the chemokines found in the DE analysis were also found in an important module, alongside other genes showing similar expression patterns that were not found in the original analysis. Correlation of such modules with external factors is also often overlooked. We have shown that transcripts and proteins for the same gene can correlate with distinct modules, posing novel hypotheses about post-translational modifications that may affect protein stability and activity.

The biological findings shown here are currently being studied further, to elucidate the role of the collagen genes, ECM and basement membranes in PMM2-CDG and the potential role of the chaperones in restarting the cell-cycle process. For Lafora disease, the relationship between the gene modules and chemokines is being investigated. These appear related to the secretion of pro-inflammatory mediators and as such the results presented here indicate novel transcriptional regulators.

In terms of how the package has been implemented, it can run as user-written scripts, combining the different functions as required, or directly from the command line, requiring little previous R knowledge. As such, the package can be used by the widest possible user-base. This command line usage also means the package can be run on computer clusters, important when running co-expression analysis on large datasets. In addition, the reports have been designed to provide intuitive explanations of the different stages of the analysis, showing multiple graphical representations of the data and how to interpret them. The modular design of the package permits the user to jump to specific steps in the methodology. We would like to emphasize the need for the stable implementations of proposed methodologies and workflows.

## Material and methods

### Package overview

The ExpHunter Suite methodology has been implemented as two main analysis modules. They can be run interactively as conventional R packages or directly from the command line as scripts. The DEgenes Hunter module performs quality control, expression-based filtering, DE and co-expression analysis. The functional Hunter module performs functional enrichment analyses, using the output of the DEgenes Hunter module as input. Both produce multiple files and reports as shown in Fig. 1. The modules can be run from the command line as scripts or from within the R environment functions. Full details at https://bioconductor.org/packages/ExpHunterSuite.

### Quality control, filtering and normalisation

The count table can be filtered to remove genes with little evidence of expression, based on a minimum number of reads mapping to a minimum number of samples (two counts per million mapped reads in two samples by default).

To assess data quality, the package runs principal component analysis (PCA), calculates correlation between samples and produces expression heatmaps, before and after normalisation. More advanced quality control is also implemented for the DEG and CEG analysis packages.

### Differential expression analysis

ExpHunter Suite can launch one or several DEG detection packages. Currently, edgeR, limma, NOISeq and DESeq2 are included^34–37^ using default parameters.

DEG detection packages require a table of counts and an indication of which samples are controls and which are treated, specified in the target file or by input arguments. Overexpressed and underexpressed genes in treated samples will have positive or negative base 2 logarithmic fold change (log2 fold change, logFC) values, respectively. The target file can also contain additional factors to include in the DE models, such as pairing and control for external factors. Columns in this file can also be included in multifactorial experimental designs, to look for group-specific changes and interactions between factors.

Genes are tagged as prevalent/possible DEGs, based on package results, using a user-specified threshold: if a gene is detected as DE by at least as many packages as the threshold, it is considered a prevalent DEG according to this vote system. Conversely, if a gene is detected as DE by at least one method but fewer than the threshold, it is considered a possible DEG. By default, the threshold comprises an adjusted p-value < 0.05 and absolute logFC $\ge$ 1.

The package also performs score integration to obtain combined logFC and adjusted p-value/FDR values for each gene across all packages. LogFC values are combined using the arithmetic mean and the FDR values are combined using Fisher’s method. The combined-FDR can also be used to decide whether a gene is DE, instead of the vote system described above.

#### Naïve Bayes classifier

The results of the different DE detection packages can also be combined using a Naïve Bayes classifier. For this approach, we train the model using vectors of p-values calculated by each package alongside labels indicating whether the vector represents a DEG or non-DEG. These vectors are derived from simulated datasets described below in the Study datasets sections. Multiple datasets were created including different numbers of genes changing between case and control samples and different magnitudes of fold change. The model, once trained on these datasets, can then be used to predict DEG status for a novel gene, given a vector of p-values. Full details are given in Supplementary Methods.

### Co-expression network analysis

Weighted gene co-expression network analysis (WGCNA)^11,38^ is employed to locate modules of genes showing correlated expression across samples. This process is automated, but produces various graphics that can indicate problems that require user intervention. The expression values for the genes in each module are also summarised to produce a single value per sample (eigen-gene value)^11^. Correlation between the eigen-gene values for each module and the additional factors in the target file are also calculated. For quantitative values, correlation is calculated directly using Pearson’s correlation coefficient. Qualitative variables are first converted into binary vectors using the WGCNA package. Full details in Supplementary Methods.

### Functional analysis

The functional analysis module is aimed at interpreting gene lists by looking for enrichment of sets of functionally related genes, i.e. sets of genes involved in the same biological process/pathway, with shared function or similar cellular location. These sets can be predefined, or the user can supply his own set using the Gene Matrix Transposed file format (*.gmt). Functional analysis using a non-model organism can be performed using an orthologue dictionary to borrow information from another species. This module integrates directly with the DEgenes Hunter module, searching for enrichment within the lists of identified DEGs/CEGs.

Multiple annotation systems have been integrated: Gene Ontology^39^, KEGG^40^ and Reactome Pathway Knowledgebase^41^. Over-Representation Analysis is used, based on significant overlap between the input DEG/CEG gene list and the different sets of functionally related genes^42^. Gene Ontology (GO) is analysed using both topGO^43^ and clusterProfiler^42^, KEGG using clusterProfiler and Reactome using ReactomePA^44^.

### Output files and results

Tables of results are generated for each of the DEG detection methods specified. A general output table is also created, which contains all DEG and CEG related results. HTML reports that allow the user to inspect the results and identify potential problems are also produced. The DE analysis report contains multiple plots for checking the quality of the samples, including Venn diagrams and bar charts of the results of the DEG detection methods. A section containing the co-expression results is also added, if applicable.

The functional analysis module produces a set of tables showing the enriched categories. There is a general report to help the reader interpret the results and identify the most relevant enriched terms, as well as connections between terms. If co-expression analysis is performed, a report is also created for each module, containing additional information such as plots of gene expression and the relationship between eigen-gene values and phenotypic data.

### Study datasets

We show the utility of our methodology by applying it to simulated datasets, publicly available spike-in data and real RNA-seq rare-disease datasets. Full details including experimental design and how the packages were used are given in Supplementary Methods. *Simulated datasets* The ExpHunter Suite includes methodology to simulate an RNA-seq counts table, based on the R package TCC^21^. Full details are given in Supplementary Methods. Using this method, we produce datasets based on the *Arabidopsis thaliana* count table from TCC and the real disease datasets. In total, 24,300 simulated datasets were produced, investigating a range of parameters (Supplementary Methods, Table 1). The AutoFlow workflow manager was used to handle package executions^45^. *Spike-in data* RNA-seq data was obtained from a previously published experiment using mouse embryonic stem cells, for which synthetic RNA corresponding to 47 transcripts had been added (spiked-in) to the samples before sequencing^46^. These transcripts correspond to endogenous mouse genes whose expression could not be detected in these samples. *Real study cases* We applied our methodology to two rare disease datasets. A minimum-vote threshold of 3 was used to determine prevalent DEGs for downstream analysis. The first disease, PMM2-CDG is a heterogeneous, multi-systemic disease caused by the deficiency of the PMM2 enzyme, for which there is no effective treatment^47,48^. The dataset was derived from skin fibroblast cell lines from patients and controls, and distinct groups of samples were derived before and after the addition of a molecular chaperone. The second, Lafora disease is a neurodegenerative disorder that leads to progressive myoclonus epilepsy, characterised by the accumulation of insoluble poorly branched glycogen deposits in the brain and peripheral tissues^49^. The dataset consisted of three groups of mice: two mutant groups which exhibited disease symptoms (*Epm2a* and *Epm2b*) and a control group.

The study was approved by the Ethics Committee of the Universidad Autonoma de Madrid (CEI-105-2052) and conducted according to the principles of the Declaration of Helsinki. All participants gave informed consent.

## Supplementary Information

Supplementary Legends.

Supplementary Information 1.

Supplementary Information 2.

Supplementary Information 3.

Supplementary Report 1.

Supplementary Report 2.

Supplementary Report 3.

Supplementary Report 4.

Supplementary Report 5.

Supplementary Report 6.

## Data Availability

Code is available at the bioconductor landing page https://bioconductor.org/packages/ExpHunterSuite. The latest version of the code can be found at https://github.com/seoanezonjic/ExpHunterSuite. There is a specific github site for the simulated data and case-studies at: https://github.com/fmjabato/ExpHunterSuiteExamples. The dataset supporting the results of this article are available in the Sequence Read Archive SRA [https://www.ncbi.nlm.nih.gov/sra/PRJNA746239 (Lafora Disease) and https://www.ncbi.nlm.nih.gov/sra/PRJNA747153 (PMM2-CDG)]; all FASTQ files as well as important processed data necessary to repeat analysis have been made available.
