# Supplementary material for: Gene expression analysis method integration and co-expression module detection applied to rare glucide metabolism disorders using ExpHunterSuite
Source: Sci Rep. 2021 Jul 23;11:15062. doi: 10.1038/s41598-021-94343-w (PMC8302605; doi:10.1038/s41598-021-94343-w)
Supplement: Supplementary file 1 — Supplementary Legends. [file 41598_2021_94343_MOESM1_ESM.docx]

**Supplementary Files**

Supplementary Methods: File containing additional description of the methods used in this study.

Supplementary Results Methods Comparison: Supplementary Figures related to the comparisons between different

DEG detection methods.

Supplementary Results Case Studies: Full details of the rare disease dataset analyses undertaken using ExpHunter

Suite.

Supplementary Report 1: HTML report with all results obtained for each simulated experiment analysed (A. thaliana,

Lafora disease and PMM2-CDG).

Supplementary Report 2: HTML report with DEG results obtained for the spike-in analysis.

Supplementary Report 3: HTML report comparing the estimated spikein logFC values obtained by the different DEG

detection methods with the known values, for each combination of mixes.

Supplementary Report 4: ExpHunter DEG report for the PMM2-CDG case study

Supplementary Report 5: ExpHunter DEG report for the Lafora disease case study.

Supplementary Report 6: ExpHunter Functional report for the Lafora disease case study, cluster 1.
