# Supplementary material for: Gene expression analysis method integration and co-expression module detection applied to rare glucide metabolism disorders using ExpHunterSuite
Source: Sci Rep. 2021 Jul 23;11:15062. doi: 10.1038/s41598-021-94343-w (PMC8302605; doi:10.1038/s41598-021-94343-w)
Supplement: Supplementary file 2 — Supplementary Information 1. [file 41598_2021_94343_MOESM2_ESM.pdf]

1 Supplementary Methods

1.1 Simulated data generator

Our simulated data generator package simulates an RNA-seq count table using a set of Arabidopsis-derived samples, generating treatment samples by modifying selected genes to be either up or down-regulated. It includes the following additional features: simulation based on user-input data properties; specify proportions of over and under-expressed genes; and usage of custom functions to fit fold-change proportions to enable the use of more realistic distribution models. With these new features we are able to simulate a pool of experiments by configuring a grid of parameters to test the DE algorithms (Table 1).

Table 1: Ranges of parameters used for simulated count table generation.

Parameter	Values
Log 2 fold change	2, 3, 4
Percentage of DEGs	0%, 1%, 5% and 10%
Ratio of up/down-regulated genes	fixed at 1:1
Number of genes in each experiment	10000, 20000, 30000
Number of replicates per sample group	3, 5, 10

1.2 Naïve Bayes Classifier

The Naïve Bayes classifier is trained using the simulated datasets for which a known amount of genes are changing in expression. The distributions of p-values for each method are modelled for DEG /non-DEG class labels.

More concretely, for each gene, the probability of being classified DEG given a vector of p-values for the different methods, X is given by the following:

$$p(DEG \mid \mathbf{x}) = \frac{p(DEG)\ p(\mathbf{x} \mid DEG)}{p(\mathbf{x})}$$

Where the prior probability is estimated from the training data.

1.3 Converting qualitative vectors to binary

Qualitative vectors were converted following the recommendations of the WGCNA author. Where the variables are qualitative, they will be transformed into multiple vectors of 0 or 1. There will be as many vectors as the

number of different factors in the original variable. Each vector will be of the same length as the number of samples and will hold the value of 1 for samples corresponding to the given factor; all other samples will represented by 0. Correlation with the treatment and control samples will always be calculated.

1.4 General analysis steps and protocol options

Before running ExpHunter Suite modules on the datasets, quality assessment was performed using FastQC and seqtrimBB based on BBtools suite [1]. For the Lafora disease case study and spike-in data, reads were aligned against the mouse genome (GRCm38), version M23 (Ensembl 98), with annotation obtained from GENCODE (2019-09-06). For the PMM2 case study, reads were aligned against the human genome (GRCh37), annotation GENCODE v19. In all cases, STAR (2.5.3a) was used for alignment to obtain the table of counts to be used as input by ExpHunter Suite [2]

To analyse the real and spike-in datasets, default ExpHunter Suite parameters were used for adjusted p-value (0.05), logFC threshold (1), and lowly-expressed gene filtering (2 counts per million mapped reads in at least 2 samples per group), with the exception of PMM2, as described below.

1.5 Spike-in dataset

The RNA species were added in mixes of three different concentrations, for genes with single transcripts per locus and with multiple transcripts per locus as shown in the supporting material: S1 Table and S2 Table from [9], corresponding to three groups of samples. A fourth group was also included, to which no spike-in transcripts were added. Four samples were used for each group, corresponding to 16 in total. Total RNA was processed using the Illumina TruSeq Strand Specific total RNA with RiboZero Gold protocol and sequenced on the Illumina HiSeq Rapid 2500 instrument, obtaining paired-end 100-bp reads. Full details of the experimental design, spike-in quantity, length and sequence, quality assessment and sequencing protocol are given in [9]. RNA-seq data was obtained from the Sequence Read Archive [8] (Study ID: SRP062126). We ran ExpHunter Suite six times, for all possible pairwise comparisons between the four groups, detecting DEGs using the four implemented DE detection methods.

1.6 PMM2-Congenital Disorder of Glycosylation

PMM2-Congenital Disorder of Glycosylation (CDG) is a heterogeneous, multisystemic disease caused by the deficiency of the enzyme phosphomannomutase 2 (PMM2), for which there is no effective treatment [10]. The molecular pathomechanisms underlying the link between the defects in this enzyme, impaired glycosylation and the clinical symptomatology are not fully understood, and little is known about the molecular basis responsible for the differences in clinical severity [10]. Unravelling these molecular and cellular pathways will help us to identify new potential therapeutic targets [3].

Skin fibroblast cell lines from three PMM2-CDG patients and three healthy control individuals were used to create a transcriptomics dataset. Cell cultures were synchronized by serum starvation to reduce bias in gene expression, following which cells were lysed and total RNA was extracted using the RNeasy Micro Kit (QIAGEN). Purified RNA samples were sequenced at the National Centre for Cardiovascular Research (CNIC, Madrid, Spain) using an Illumina HiSeq 2500 platform. To filter lowly-expressed genes, 1.5 counts per million mapped reads in at least 2 samples per group were required.

1.7 Real study case: Lafora Disease

Lafora disease is a neurodegenerative disorder that leads to progressive myoclonus epilepsy, characterized by the accumulation of insoluble poorly branched glycogen deposits in the brain and peripheral tissues [5]. These are caused by mutations in either the *EPM2A* gene, encoding the glucan phosphatase laforin, or the *EPM2B* gene, encoding the E3-ubiquitin ligase malin, leading to polyglucosan formation [4, 6].

The RNA-Seq dataset was produced using animal models of Lafora disease: *Epm2a*$^{-/-}$ mice, lacking exon 4 from the *Epm2a* gene [4], and *Epm2b*$^{-/-}$ mice, lacking the single exon present in the *Epm2b* gene [6]. Whole brain samples were taken from mice at 16 months of age. RNA was obtained as described in [7] and quality was evaluated using RNA 6000 Nano kit and Agilent 2100 Bioanalyzer System (Agilent, Madrid, Spain). The RNA-seq experiment was conducted by the Multigenic Analysis Unit from the UCIM-INCLIVA (University of Valencia, Valencia Spain) and libraries were sequenced on the Illumina NextSeq 550 to generate 75-bp single read. Four *Epm2a*$^{-/-}$, three *Epm2b*$^{-/-}$ and four control male mice were analysed. Full details can be found in [7].

Ethics approval and consent to participate

The study was approved by the Ethics Committee of the Universidad Autónoma de Madrid (CEI-105-2052) and conducted according to the principles of the Declaration of Helsinki. All participants gave informed consent.

References

[1] B Bushnell. BBTools: A Suite of Fast, Multithreaded Bioinformatics Tools Designed for Analysis of DNA and RNA Sequence Data. *Joint Genome Institute: Berkeley, CA, USA, 2018*, 2019.

[2] Alexander Dobin, Carrie A Davis, Felix Schlesinger, Jorg Drenkow, Chris Zaleski, Sonali Jha, Philippe Batut, Mark Chaisson, and Thomas R Gingeras. STAR: ultrafast universal RNA-seq aligner. *Bioinformatics (Oxford, England)*, 29(1):15–21, jan 2013.

[3] Alejandra Gámez, Mercedes Serrano, Diana Gallego, Alicia Vilas, and Belén Pérez. New and potential strategies for the treatment of PMM2-CDG. *Biochimica et Biophysica Acta - General Subjects*, 1864(11), nov 2020.

[4] Subramaniam Ganesh, Antonio V. Delgado-Escueta, Toshiro Sakamoto, Maria Rosa Avila, Jesus Machado-Salas, Yoshinobu Hoshii, Takumi Akagi, Hiroshi Gomi, Toshimitsu Suzuki, Kenji Amano, Kishan Lal Agarwala, Yuki Hasegawa, Dong Sheng Bai, Tokuhiro Ishihara, Tsutomu Hashikawa, Shigeyoshi Itohara, Eain M. Cornford, Hiroaki Niki, and Kazuhiro Yamakawa. Targeted disruption of the Epm2a gene causes formation of Lafora inclusion bodies, neurodegeneration, ataxia, myoclonus epilepsy and impaired behavioral response in mice. *Human Molecular Genetics*, 2002.

[5] Maria García-Gimeno, Erwin Knecht, and Pascual Sanz. Lafora Disease: A Ubiquitination-Related Pathology. *Cells*, 7(8):87, jul 2018.

[6] C. Gómez-Abad, P. Gómez-Garre, E. Gutiérrez-Delicado, S. Saygi, R. Michelucci, C. A. Tassinari, S. Rodríguez De Córdoba, and J. M. Serratosa. Lafora disease due to EPM2B mutations: A clinical and genetic study. *Neurology*, 2005.

[7] Marcos Lahuerta, Daymé Gonzalez, Carmen Aguado, Alihamze Fathinajafabadi, José Luis García-Giménez, Mireia Moreno-Estellés, Carlos

Romá-Mateo, Erwin Knecht, Federico V. Pallardó, and Pascual Sanz. Reactive Glia-Derived Neuroinflammation: a Novel Hallmark in Lafora Progressive Myoclonus Epilepsy That Progresses with Age. *Molecular Neurobiology*, 2020.

[8] Rasko Leinonen, Hideaki Sugawara, and Martin Shumway. The sequence read archive. *Nucleic Acids Research*, 2011.

[9] Dena Leshkowitz, Ester Feldmesser, Gilgi Friedlander, Ghil Jona, Elena Ainbinder, Yisrael Parmet, and Shirley Horn-Saban. Using synthetic mouse spike-in transcripts to evaluate RNA-seq analysis tools. *PLoS ONE*, 2016.

[10] Patricia Yuste-Checa, Alejandra Gámez, Sandra Brasil, Lourdes R. Desviat, Magdalena Ugarte, Celia Pérez-Cerdá, and Belén Pérez. The Effects of PMM2-CDG-Causing Mutations on the Folding, Activity, and Stability of the PMM2 Protein. *Human Mutation*, 2015.
