# Supplementary material for: Gene expression analysis method integration and co-expression module detection applied to rare glucide metabolism disorders using ExpHunterSuite
Source: Sci Rep. 2021 Jul 23;11:15062. doi: 10.1038/s41598-021-94343-w (PMC8302605; doi:10.1038/s41598-021-94343-w)
Supplement: Supplementary file 3 — Supplementary Information 2. [file 41598_2021_94343_MOESM3_ESM.pdf]

1 Supplementary Results - Comparison between methods

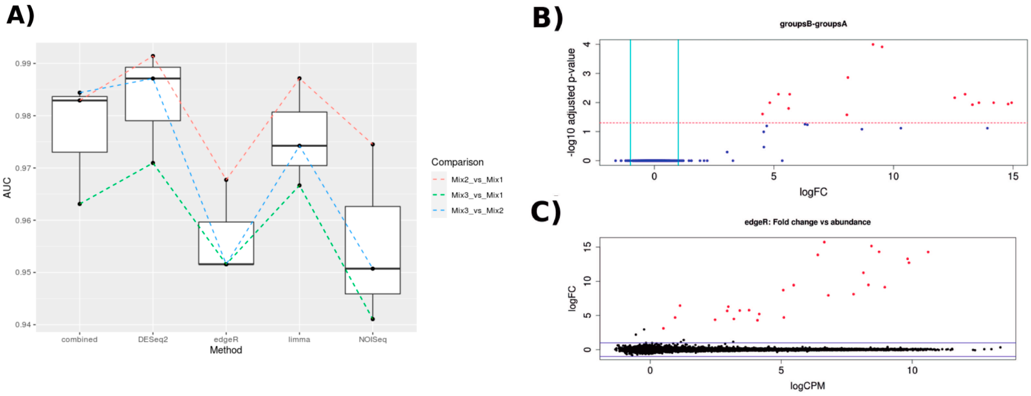

Figure 1: **Performance of the different DEG detection methods and vote system to detect DEGs.** A) AUC values for each individual method. Data points are connected according to the comparison between spike-ins for which they were calculated. B) Results of the limma package for the comparison Mix3 vs. none, showing log 2 fold change vs. adjusted p-value. Red points refer to significant DEGs. C) Results of edgeR for the same comparison, showing gene expression vs. log 2 fold change. Red points refer to significant DEGs. Plots B and C taken directly from the ExpHunter Suite output report.

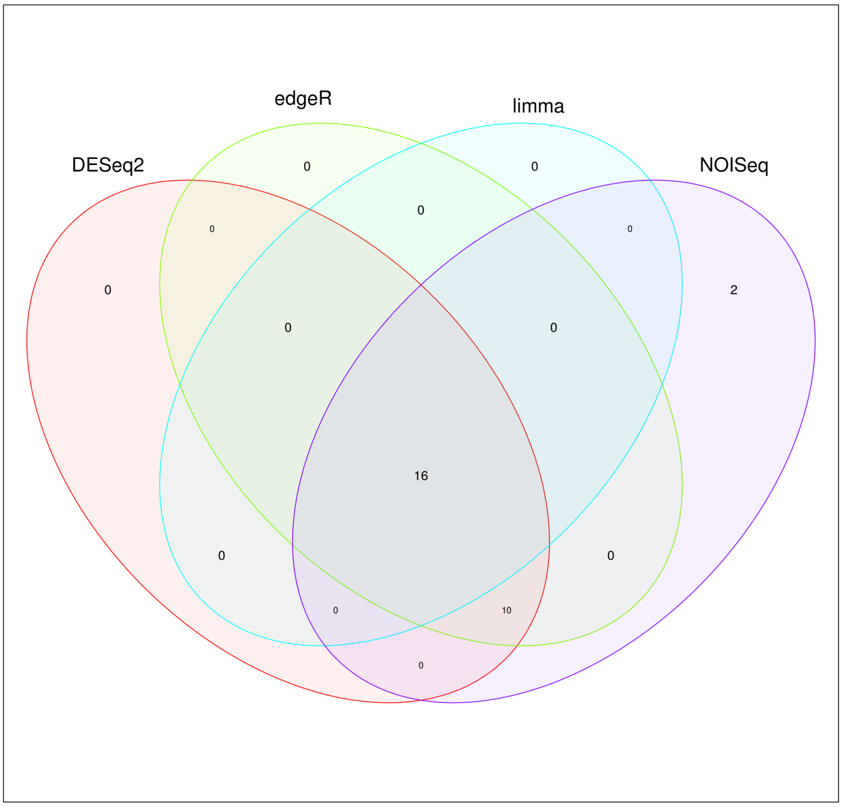

Figure 2: **Venn diagram showing the overlap in genes determined to be DE for the comparison between spike-in Mix3 and no added spike-in**
