# Supplementary material for: Gene expression analysis method integration and co-expression module detection applied to rare glucide metabolism disorders using ExpHunterSuite
Source: Sci Rep. 2021 Jul 23;11:15062. doi: 10.1038/s41598-021-94343-w (PMC8302605; doi:10.1038/s41598-021-94343-w)
Supplement: Supplementary file 4 — Supplementary Information 3. [file 41598_2021_94343_MOESM4_ESM.pdf]

Real dataset analysis

PMM2-CDG

PCA analysis showed clear separation between patient and control samples, and that the control samples present greater variability (Figure 1A). Notably, chaperone treatment appears to have little effect.

DEG detection analysis comparing patients and controls found 1081 DEGs detected by at least one method, of which only 345 were detected by all three, with DESeq2 deeming many more genes DE than the others (Figure 1B, Supplementary Report 4). Functional analysis found pathways related to muscle contraction, RHO GTPase activity, the extracellular matrix (ECM) and collagen related processes, connected by shared genes (Figure 1C). The genes tended to be underexpressed, with the notable exception of *COL14A1*.

These results support the alteration of the formation and composition of basement membranes, a specialised layer underlying multiple cell types including peripheral nerve axons and adipocytes [13]. Collagen type IV genes, as well as laminins, nidogen 1 and 2, and several proteoglycans are key to these processes. The reduction of *COL4A1* and *COL4A2* expression in the PMM2–CDG samples is of note. These genes impair collagen IV network formation due to aberrant collagen structure and have been associated with intra-cerebral haemorrhages and stroke–like episodes, and defects in the retinal vasculature and glomerular basement membrane [1, 2], both found in PMM2-CDG. Moreover, they may be exacerbated by endoplasmic reticulum stress, a hallmark of CDGs [10, 14]. The increase in *COL14A1* may be a response to collagen type IV down-regulation.

Transmembrane proteins involved in ECM structure are also affected, including integrins like *ITGA3*, and receptors for paracrine signals (both *WNT5A* and its receptor *FZD2* are down regulated). Furthermore, pathways responsible for cytoskeleton dynamics seem to be involved, related to semaphorins and Rho GTPases, suggesting that cell migration and proliferation may be compromised. Nevertheless, skin–derived fibroblasts have limitations in modelling PMM2-CDG and these hypotheses must be further validated.

Co-expression analysis was performed to find modules correlated with patient samples, individual samples and the presence of chaperones. The individual_ctl_1 sample is close to co-expression module 2 (correlation=0.98, p-value=7×10^{-8}) (Figure 3A). This module contains 843 genes of which only 5 are prevalent DEGs. The genes in this module tend to show higher expression in samples ctrl1_Chap and ctrl_noChap (Figure 2A). As such, these genes can be considered individual-specific. The ability of our methodology

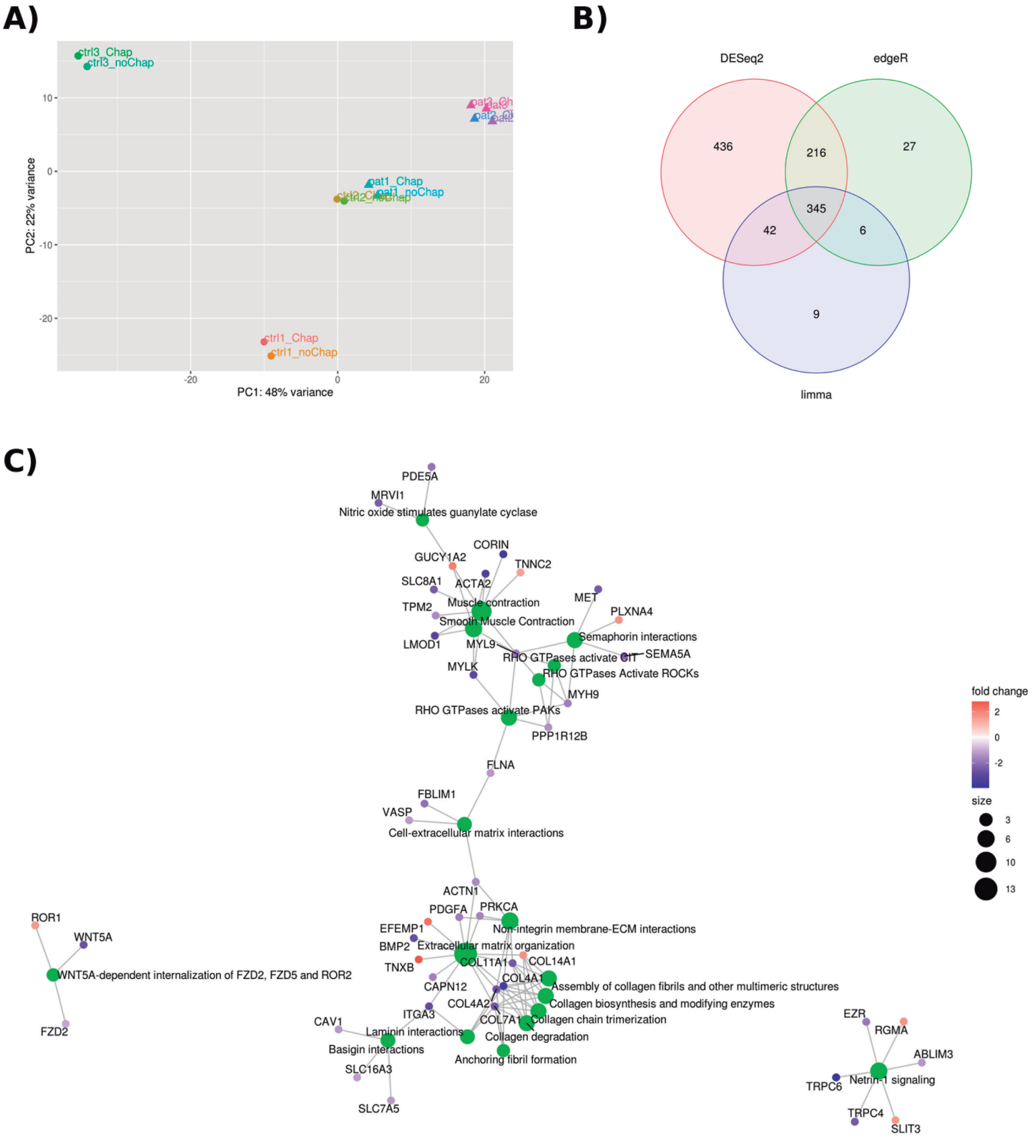

Figure 1: **Results for the analysis of the PMM2-CDG dataset.** A) PCA showing the separation of samples along the first two principal components. The greatest amount of variance was found between control and patient samples, although the Ctrl_2 and pat_1 samples were found close together. B) Venn diagram showing the results of the three different DEG detection methods employed. C) Representative functional enrichment plot for GO Biological Processes. All figures taken directly from the ExpHunter Suite output report.

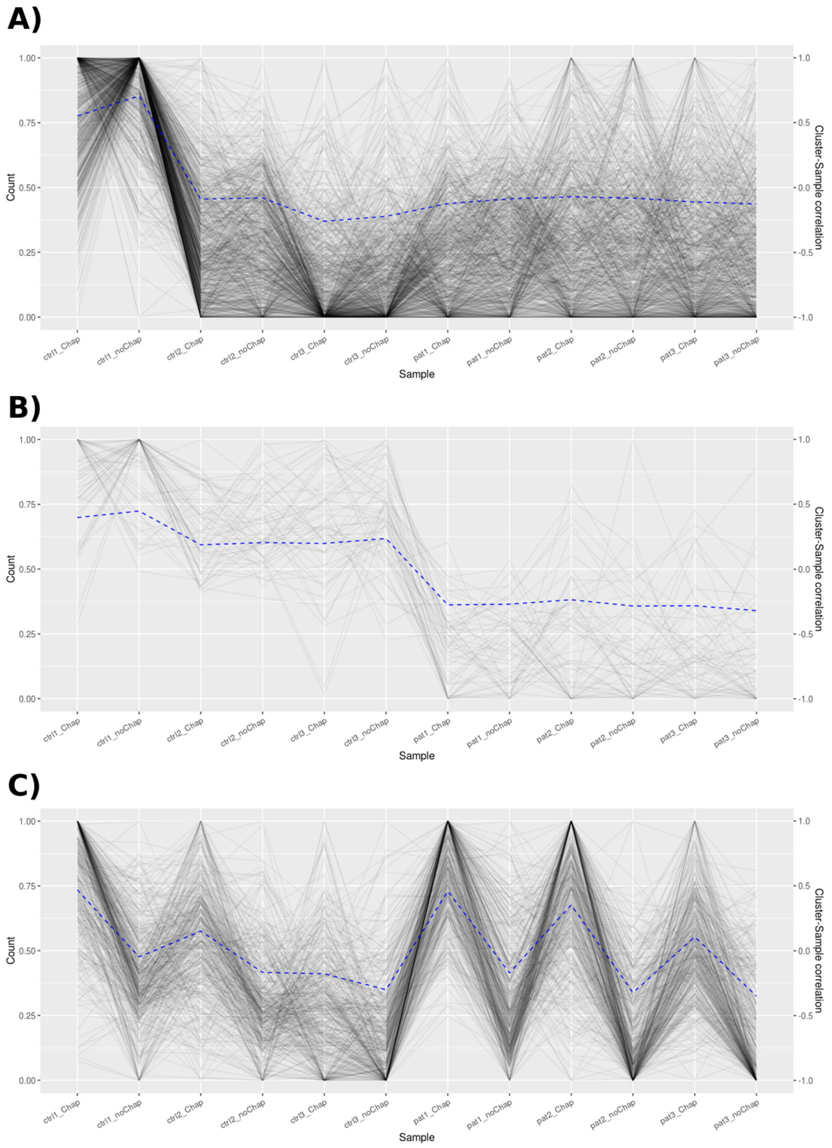

Figure 2: **Co-expression profiles for A) Module 2 B) Module 60 C) Module 6 found for PMM2–CDG.** X-axis shows the different samples; y-axis shows the expression values, normalized to the range 0:1; lines represent values for different genes within a given module. Blue line represents the eigen-gene for that module.

to identify such individual-specific genes is important as they allow the user to find groups of genes that can be potentially excluded from downstream analysis.

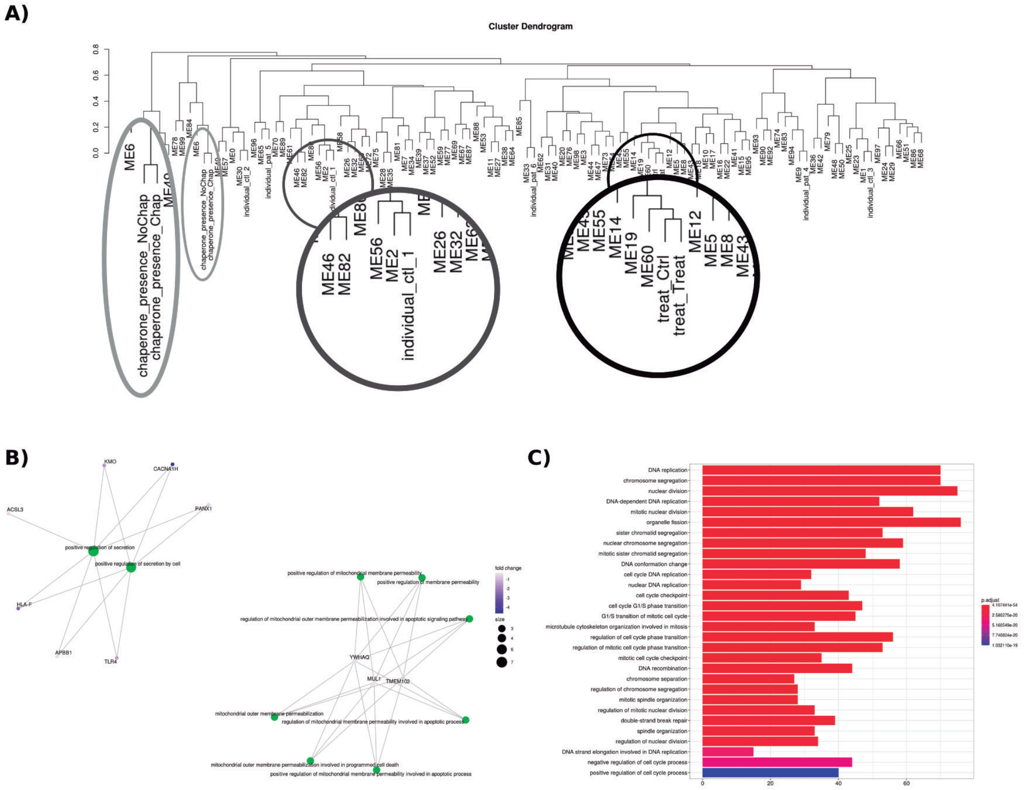

Figure 3: **Co-expression results for the analysis of the PMM2-CDG dataset.** A) Dendrogram based on correlation between the co-expression modules and the categorical vectors. B) Function-gene network representation of the GO Biological Process enrichment for the co-expression module 60, the closest module to the treat-control vectors C) Barplot representing enrichment for the co-expression module 6, the closest to the chaperone vectors. The data is shown in this format as the network representation was too dense to interpret due to the large numbers of categories and genes.

In terms of correlation with the treated samples, the vectors encoding this information are close to module 60 (correlation=0.97, p-value=4×10^{-7}) This module contains 58 genes of which 12 are prevalent DEGs. The genes in this module show higher expression in the patient samples (Figure 2B). Enrichment analysis shows reduced expression for genes related to cell secretion, mitochondrial membrane permeability, and apoptotic processes (Figure 3B).

CACNA1H is underexpressed by 4.6 logFC. This gene encodes the voltage–gated calcium channel Cav3.2. Notably, another calcium channel, Cav2.1,

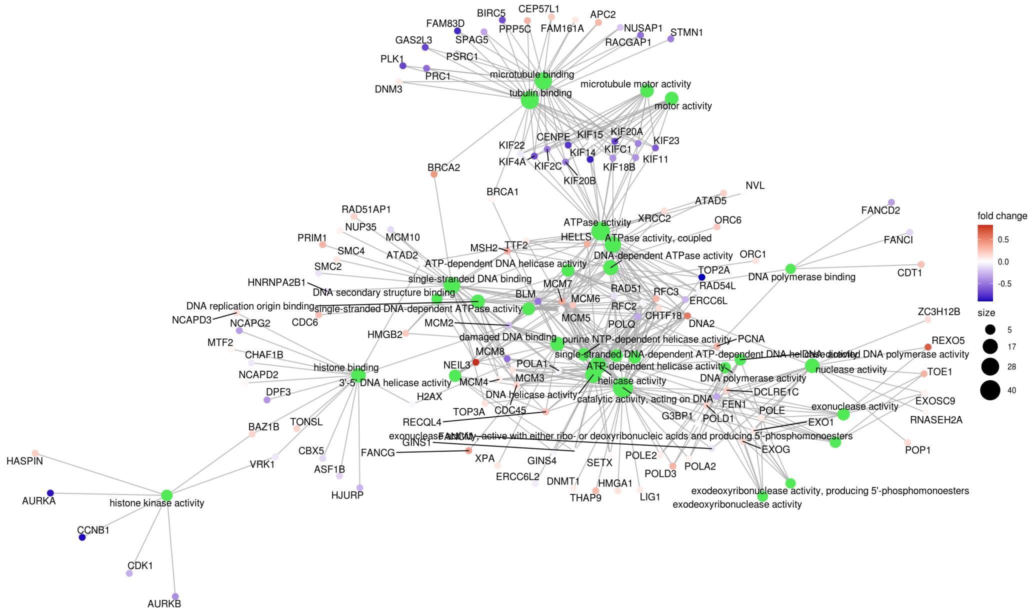

Figure 4: **GO Molecular Function network for co-expression module 6.** Green points represent functional categories, blue/red points represent genes belonging to these categories, with the colour indicating fold-change between patients and controls. LogFC values range from -0.5 to 0.5, the most underexpressed genes are *TOP2A*, *AURKA* and *CCNB1* whereas *NEIL3*, *REXO5* and *DNA2* are the most overexpressed. There are also several Kinesins genes with low expression.

encoded by *CACNA1A*, has been reported to be involved in PMM2-CDG cerebellar syndrome and has been proposed as a therapeutic target [6]. *CACNA1H* loss of function mutations and the consequent decreased activity of the Cav3.2 channel have been related to development of autism spectrum disorder [11], and its decreased expression has been linked to skeletal muscle atrophy involving endoplasmic reticulum stress.

Regarding the addition of a chaperone to the samples, module 6 was closest to the vector encoding this information (Figure 3A; correlation=0.79, p-value=0.002). The genes in this module tend to show higher expression in the chaperone receiving samples than controls (Figure 2C), moreover the pattern is much clearer for patient samples, as would be expected given that the chaperone is intended to rescue the function of the mutated *PMM2* gene. The genes in this module are enriched for various GO terms related to the cell cycle and DNA processing (Figures 3C and 4). Taken together, these results suggest that the chaperone may be helping to restore these processes.

Lafora disease

For the Lafora disease dataset, PCA analysis revealed separation between control and knock-out samples (*Epm2a* and *Epm2b*) along the first principal component, and between *Epm2a* and *Epm2b* samples along the second, suggesting that the different mutants lead to a similar expression profile, with some differences (Figure 5A).

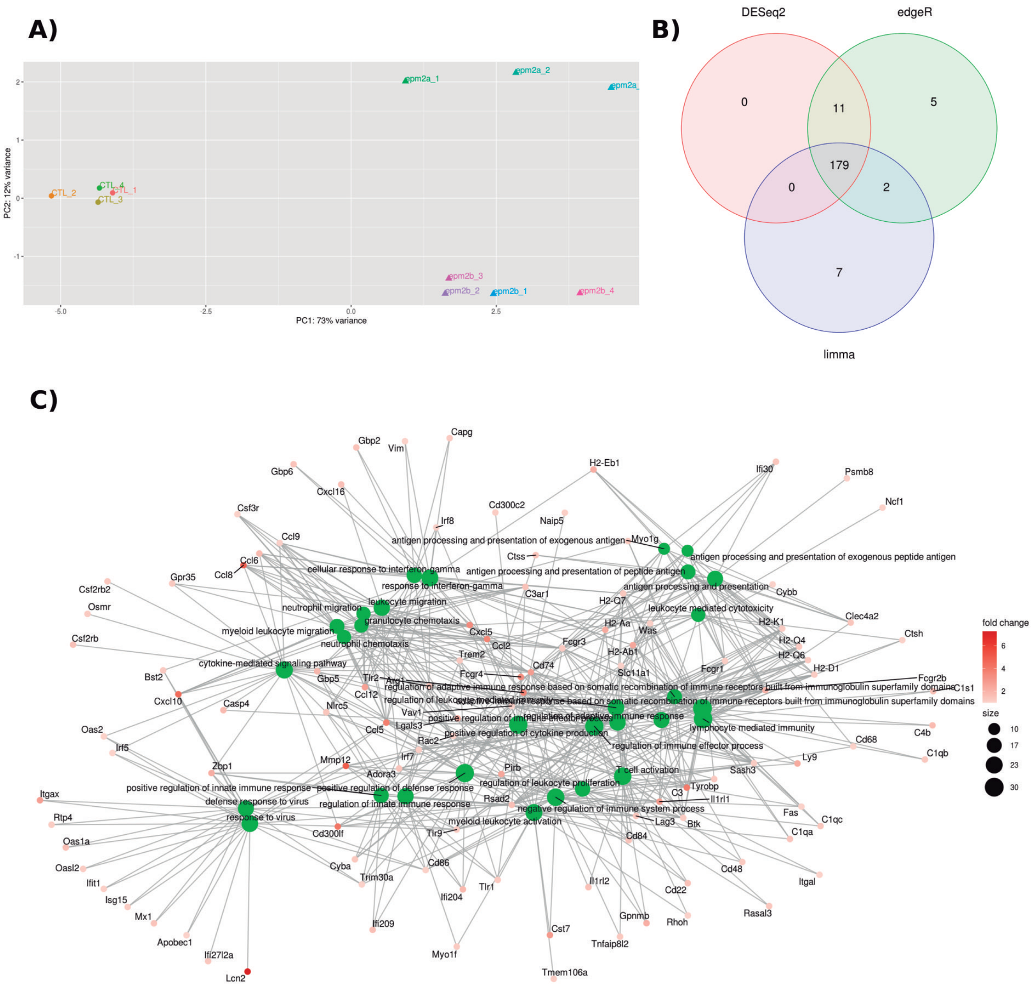

Figure 5: **Results for the analysis of the Lafora disease dataset.** A) PCA showing the separation of samples along the first two principal components. B) Venn diagram showing the results of the three different DEG detection methods. C) Representative functional enrichment plot for GO Biological Processes. All figures taken directly from the ExpHunter Suite output report.

Of the genes detected by at least one of the DEG detection methods, 179 were detected by all three packages (Figure 5B). In contrast to the results obtained for the PMM2-CDG dataset, DESeq2 did not detect any genes not detected by at least one other method (Supplementary Report 5).

Functional analysis identified genes related to the innate and adaptive immune responses and inflammation (Figure 5C), supporting the idea of microglia-astrocyte cross talk in neurodegeneration [5]. Many genes underlying these functions are highly overexpressed (Figure 5C), including *Lcn2*, that can protect the nervous system in response to inflammatory processes [3]. The up-regulated genes are largely expressed by astrocytes and microglia, two cell types that accumulate polyglucosan inclusions [8]. As such, one can speculate that this accumulation triggers pro-inflammatory mediator production in these cells [5].

Correlation analysis was first performed to find modules correlated with all mutant mice, and then focussing on specific knock-out groups. The full dendrogram can be visualised in Figure 6.

Module 1 is correlated with the mutant vs. control vectors (correlation=0.91, p-value=6.25×10^{-5}, Figure 8A). The expression values of the genes in this module can be seen in Figure 7A, tending towards higher expression in the mutant samples. These genes show enrichment for the regulation of immune responses, including negative regulation of immune effector process, positive regulation of cytokine production and T cell activation (Figure 8B). Full details are included in Supplementary Report 6.

The gene-function network for this module can not be easily visualised due to its large size. Module 13, which is a similar distance from the mutant/control vector (correlation=0.95, p-value=5.2×10^{-6}), is shown instead (Figure 9). It shows immune system and inflammatory processes, including response to virus, cellular response to lipopolysaccharide and biotic stimulus, and I-κB kinase/NF-κB signaling. All genes are overexpressed in this group, interestingly three of the most highly expressed are *Cxcl10*, *Ccl5* and *Ccl12*, also identified in the initial functional analysis.

Regarding correlation with the different knock-out mice, the *Epm2a* vector was close to module 23 (correlation=0.93, p-value=3.2×10^{-5}). This module shows higher gene expression in *Epm2a* knock-out samples (Figure 7B) and enrichment for the stress-activated MAPK cascade, JNK cascade and immune system processes (Figure 8B), consistent with glial signaling in this disease [9]. Module 23 contains *Tlr4* and *Traf6*, two components of the key inflammation pathway that triggers NF-κB and MAPK processes, important in Lafora disease [9]. *Epm2b* was highly correlated with module 11 (correlation value=0.92; p-value=5.3×10^{-5}), which contains 138 genes, none of which are DE, despite higher expression in the *Epm2a* samples (Fig-

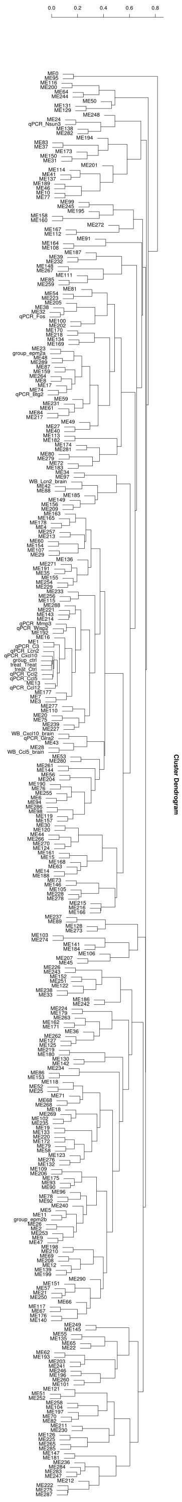

Figure 6: **Full dendogram for the Lafora disease co-expression analysis.**

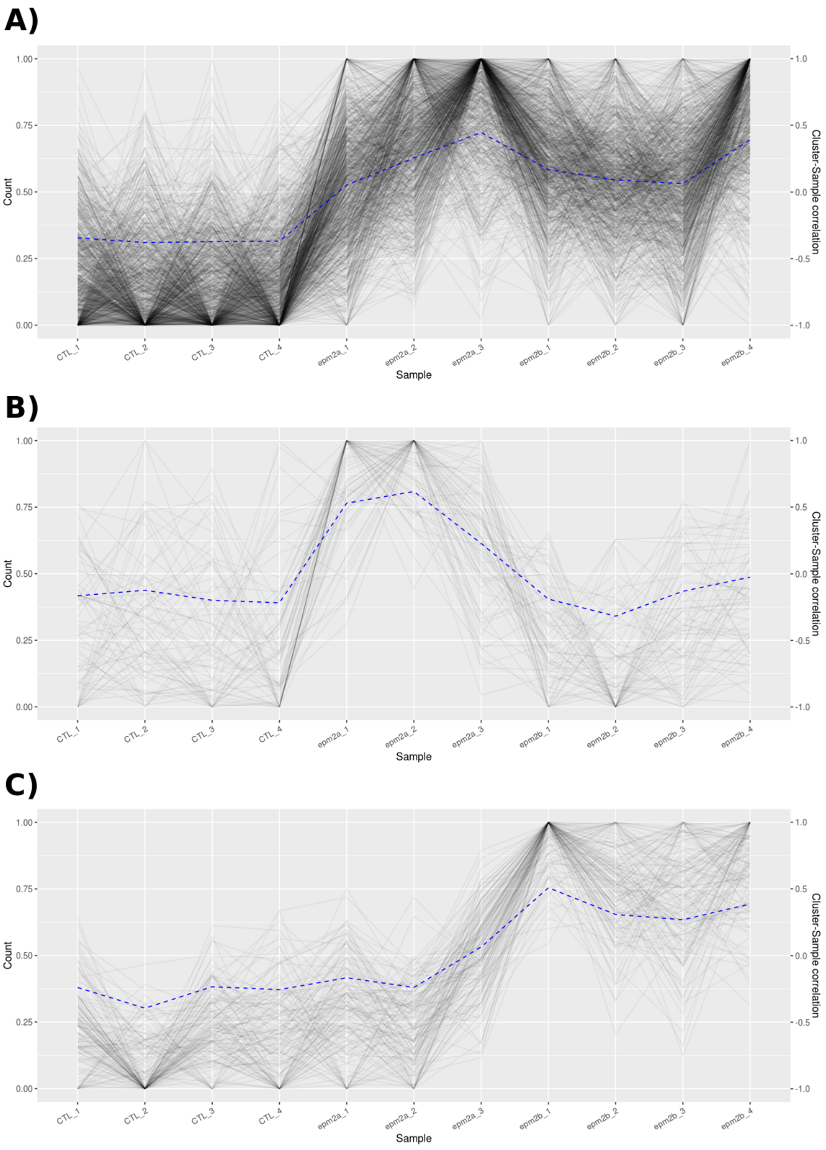

Figure 7: **Co-expression profiles for A) Module 1, B) Module 23 and C) Module 11 found for Lafora disease.** X-axis shows the different samples; y-axis shows the expression values, normalized to the range 0:1; lines represent values for different genes within a given module. Blue line represents the eigen-gene for that module.

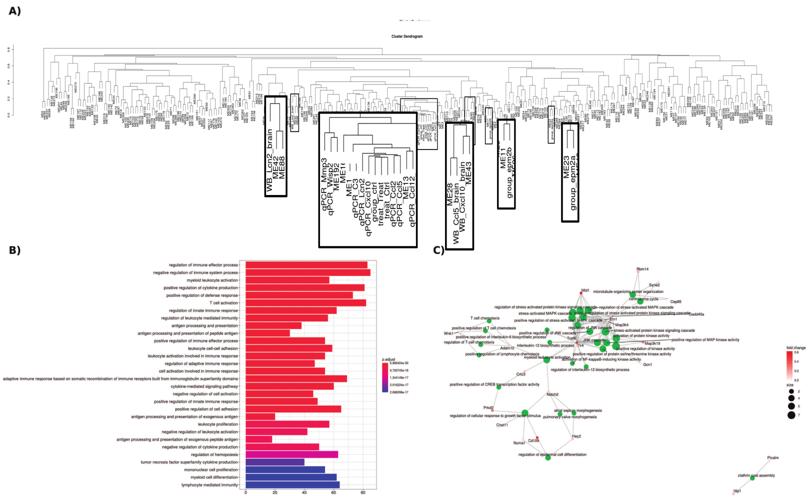

Figure 8: **Co-expression results for the analysis of the Lafora disease dataset**. A) Dendrogram based on the correlation between the co-expression modules and the categorical vectors. B) Barplot with the enrichment results using GO Biological Process for the 999 genes within co-expression module 1, the closest to the treatment/control categorical vectors. X-axis shows the number of genes ascribed the given GO term. C) Gene-function network that represents the GO Biological Process enrichment analysis for co-expression module 23.

ure 7C).

We also investigated the correlation between gene modules and gene and protein expression values, chosen based on the results of the initial DE analysis and measured using western blot (WB) and real time quantitative PCR (qPCR) (Table 1). Full details in [5]. All expressed genes in this table were detected as differentially expressed between knock-out and control animals according to all DE gene detection and combination methods, including the Naïve Bayes approach.

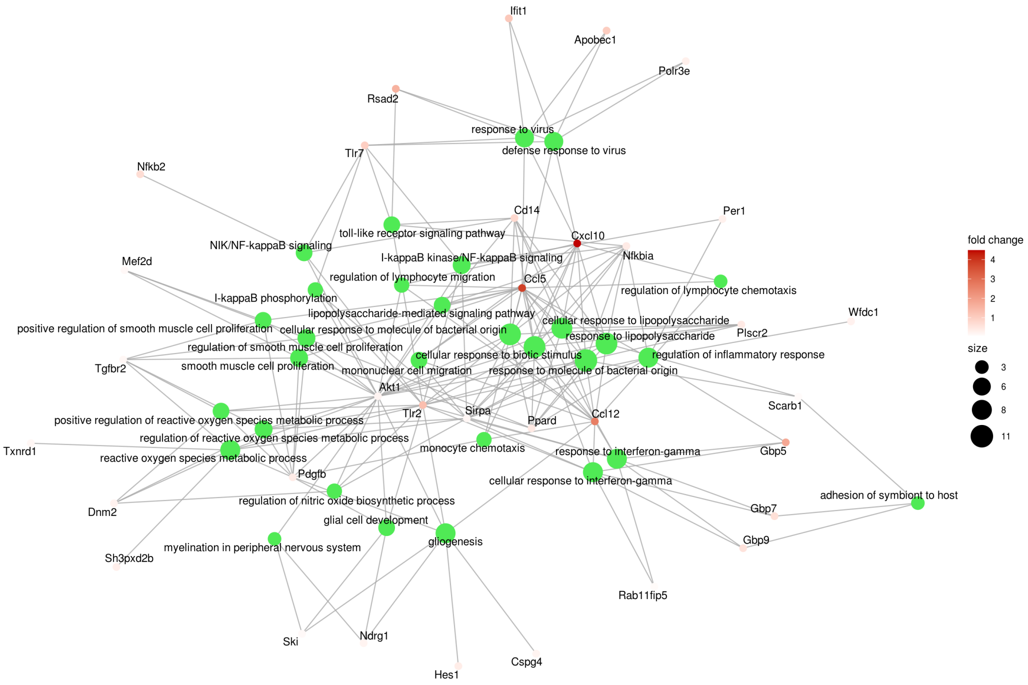

Figure 9: **Function-gene network that represents the GO Biological Process enrichment analysis for the co-expression module 13**, the next closest to the categorical vectors, for Lafora disease.

Table 1: Description of external variables for Lafora disease.

Measure	Description	Cluster	Correlation	P-value
Lcn2 (mRNA)	Lipocalin 2, an iron trafficking protein involved in neuroinflammation	ME13/ME1	0.96/0.95	$4\times10^{-6}/1\times10^{-5}$
Cxcl10 (mRNA)	C-X-C motif chemokine 10, a proinflammatory chemokine involved in chemotaxis	ME13/ME1	0.97/0.93	$9\times10^{-7}/2\times10^{-5}$
C3 (mRNA)	Complement C3 protein, involved in the activation of the complement system	ME1	0.98	8×10^{-8}
Ccl2 (mRNA)	C-C motif chemokine 2, involved in the chemotactic response and mobilization of calcium ions	ME1/ME13	0.96/0.94	$1\times10^{-6}/1\times10^{-5}$
Ccl5 (mRNA)	C-C motif chemokine 5, a chemoattractant for blood monocytes, memory T-helper cells, and eosinophils	ME1/ME13	0.96/0.95	$2\times10^{-6}/8\times10^{-6}$
Ccl12 (mRNA)	C-C motif chemokine 12, a potent chemokine acting through the CCR2 receptor	ME13	0.97	6×10^{-7}
Mmp3 (mRNA)	Matrix metalloprotease 3, a protease that degrades proteins from the extracellular matrix	ME192	-0.9	1×10^{-4}
Wisp2 (mRNA)	Wnt1-inducible-signaling protein 2, involved in the modulation of bone turnover	ME192	-0.9	1×10^{-4}
Lcn2 (protein)	Lipocalin 2, an iron trafficking protein involved in neuroinflammation	ME42/ME88	0.77/-0.76	$5\times10^{-3}/6\times10^{-3}$
Ccl5 (protein)	C-C motif chemokine 5, a chemoattractant for blood monocytes, memory T-helper cells, and eosinophils	ME28	0.94	1×10^{-5}
Cxcl10 (protein)	C-X-C motif chemokine 10, a proinflammatory chemokine involved in chemotaxis	ME43	-0.84	1×10^{-3}

qPCR derived gene expression values for *Lcn2*, *Cxcl10*, *Ccl2*, *Ccl5*, *C3* and *Ccl12* are grouped with the mutant vs. control vector in the dendrogram in Figure 8A. Figure 10, shows how these external measures tend to increase for the mutant samples but stay low for the controls. *Wisp2* and *Mmp3* gene expression values are most highly correlated with module 192, which shows enrichment for functional categories such as the Toll-like receptor signaling pathway, also involved in NF-κB and MAPK activation.

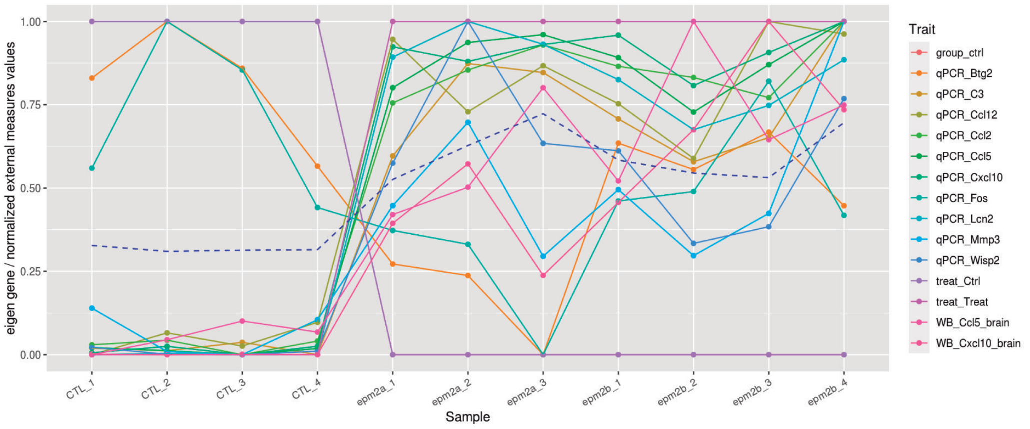

Figure 10: **Co-expression analysis results for the Lafora disease dataset using the external measures: module 1**. The eigen-gene values for module 1 are shown alongside external measure values for all significantly correlated categorical and continuous vectors (p-value < 0.05). X-axis shows different samples; y-axis shows normalised values for external variables (solid lines) and eigen-gene values (dashed blue line).

LCN2 protein levels correlate with module 42, which is enriched for the inner mitochondrial membrane protein complex. Mitochondrial activity has been shown to be altered in Lafora disease, as such the genes in this module are of interest for future study [4, 12, 7]. It should be noted that the protein levels for *Ccl5*, *Lcn2*, and *Cxcl10* correlate with different modules to the mRNA levels of their genes.

References

[1] Douglas B. Gould, F. Campbell Phalan, Saskia E. Van Mil, John P. Sundberg, Katayoun Vahedi, Pascale Massin, Marie Germaine Bousser, Peter Heutink, Jeffrey H. Miner, Elisabeth Tournier-Lasserve, and Simon W.M. John. Role of COL4A1 in small-vessel disease and hemorrhagic stroke. *New England Journal of Medicine*, 2006.

[2] Marion Jeanne, Cassandre Labelle-Dumais, Jeff Jorgensen, W. Berkeley Kauffman, Grazia M. Mancini, Jack Favor, Valerie Valant, Steven M. Greenberg, Jonathan Rosand, and Douglas B. Gould. COL4A2 mutations impair COL4A1 and COL4A2 secretion and cause hemorrhagic stroke. *American Journal of Human Genetics*, 2012.

[3] S. S. Kang, Y. Ren, C. C. Liu, A. Kurti, K. E. Baker, G. Bu, Y. Asmann, and J. D. Fryer. Lipocalin-2 protects the brain during inflammatory conditions. *Molecular Psychiatry*, 2018.

[4] Marcos Lahuerta, Carmen Aguado, Pablo Sánchez-Martín, Pascual Sanz, and Erwin Knecht. Degradation of altered mitochondria by autophagy is impaired in Lafora disease. *FEBS Journal*, 2018.

[5] Marcos Lahuerta, Daymé Gonzalez, Carmen Aguado, Alihamze Fathinajafabadi, José Luis García-Giménez, Mireia Moreno-Estellés, Carlos Romá-Mateo, Erwin Knecht, Federico V. Pallardó, and Pascual Sanz. Reactive Glia-Derived Neuroinflammation: a Novel Hallmark in Lafora Progressive Myoclonus Epilepsy That Progresses with Age. *Molecular Neurobiology*, 2020.

[6] Antonio F. Martínez-Monseny, Mercè Bolasell, Susana Callejón-Póo, ..., Jordi Muchart, Montserrat Morales, and Noemi Conde-Lorenzo. AZATAX: Acetazolamide safety and efficacy in cerebellar syndrome in PMM2 congenital disorder of glycosylation (PMM2-CDG). *Annals of Neurology*, 2019.

[7] Carlos Romá-Mateo, Carmen Aguado, José Luis García-Giménez, José Santiago Ibáñez-Cabellos, Marta Seco-Cervera, Federico V. Pallardó, Erwin Knecht, and Pascual Sanz. Increased Oxidative Stress and Impaired Antioxidant Response in Lafora Disease. *Molecular Neurobiology*, 2015.

[8] Carla Rubio-Villena, Rosa Viana, Jose Bonet, Maria Adelaida Garcia-Gimeno, Marta Casado, Miguel Heredia, and Pascual Sanz. Astrocytes: New players in progressive myoclonus epilepsy of Lafora type. *Human Molecular Genetics*, 2018.

[9] Pascual Sanz and Maria Adelaida Garcia-Gimeno. Reactive glia inflammatory signaling pathways and epilepsy, jun 2020.

[10] Jie Shang, Christian Körner, Hudson Freeze, and Mark A. Lehrman. Extension of lipid-linked oligosaccharides is a high-priority aspect of

the unfolded protein response: Endoplasmic reticulum stress in Type I congenital disorder of glycosylation fibroblasts. *Glycobiology*, 2002.

[11] Igor Splawski, Dana S. Yoo, Stephanie C. Stotz, Allison Cherry, David E. Clapham, and Mark T. Keating. CACNA1H mutations in autism spectrum disorders. *Journal of Biological Chemistry*, 2006.

[12] Mamta Upadhyay, Saloni Agarwal, Pratibha Bhadauriya, and Subramaniam Ganesh. Loss of laforin or malin results in increased Drp1 level and concomitant mitochondrial fragmentation in Lafora disease mouse models. *Neurobiology of Disease*, 2017.

[13] Yuexin Wu and Gaoxiang Ge. Complexity of type IV collagens: From network assembly to function, 2019.

[14] Patricia Yuste-Checa, Alejandra Gámez, Sandra Brasil, Lourdes R. Desviat, Magdalena Ugarte, Celia Pérez-Cerdá, and Belén Pérez. The Effects of PMM2-CDG-Causing Mutations on the Folding, Activity, and Stability of the PMM2 Protein. *Human Mutation*, 2015.
