# Supplementary material for: Gene expression analysis method integration and co-expression module detection applied to rare glucide metabolism disorders using ExpHunterSuite
Source: Sci Rep. 2021 Jul 23;11:15062. doi: 10.1038/s41598-021-94343-w (PMC8302605; doi:10.1038/s41598-021-94343-w)
Supplement: Supplementary file 5 — Supplementary Report 1. [file 41598_2021_94343_MOESM5_ESM.html]

synth\_exp\_results.utf8


# **Document summary**

This document has been generated for paper (Differential expression, co-expression and functional analysis of RNA-seq data using the DEgenes Hunter suite and its applicability to rare disease):

Measures are calculated with following formulas:

- Accuracy (ACC): \(\frac{TP+TN}{TP+TN+FP+FN}\)
- Precission (PPV): \(\frac{TP}{TP+FP}\)
- Recall: \(\frac{TP}{TP + FN}\)
- Specificity: \(\frac{TN}{TN + FP}\)
- F-Measure: \(2\*\frac{Precision \* Recall}{Precision + Recall}\)

# **Synthetic results summary**

Source table with all results is shown with a set of graphics which check the effect, over the AUC of each package, of each configured parameter. Note: samples are mixed in graphs which are not x.axis=Sample.

Also, a sumamry of all datasets are compared bellow fixing the number of genes to minimum and number of replicates to minimum, showing a grid with samples (rows) and DEG proportions (columns) and a plot of AUC obtained (y-axis) depending on the maximum fold-change simulated (x-axis) and grouping by package results (colors):

Finally, a comparisson between Vote and Combined systems is shown using different measures (rows) into each sample (columns), also two dashed lines have been added at mean of combined and largest cut of both systems:

# **Sample A. thaliana**

Results for especific sample (A. thaliana).

Summary for this sample:

```
## Warning in instance$preRenderHook(instance): It seems your data is too big
## for client-side DataTables. You may consider server-side processing: https://
## rstudio.github.io/DT/server.html
```

## **Extended study : A. thaliana**

Per each configuration, a plot of AUC (y) grouped by maximum logFC to simulate (x) will be plotted, grouping by number of genes (rows) and DEG proportion (columns) simulated:

## **Vote systems results : A. thaliana**

Measures (y) comparisson for all possible vote systems thresholds (x) grouping by number of replicates (columns) and each configuration (grid set):

# **Sample Lafora disease**

Results for especific sample (Lafora disease).

Summary for this sample:

```
## Warning in instance$preRenderHook(instance): It seems your data is too big
## for client-side DataTables. You may consider server-side processing: https://
## rstudio.github.io/DT/server.html
```

## **Extended study : Lafora disease**

Per each configuration, a plot of AUC (y) grouped by maximum logFC to simulate (x) will be plotted, grouping by number of genes (rows) and DEG proportion (columns) simulated:

## **Vote systems results : Lafora disease**

Measures (y) comparisson for all possible vote systems thresholds (x) grouping by number of replicates (columns) and each configuration (grid set):

# **Sample PMM2-CDG**

Results for especific sample (PMM2-CDG).

Summary for this sample:

```
## Warning in instance$preRenderHook(instance): It seems your data is too big
## for client-side DataTables. You may consider server-side processing: https://
## rstudio.github.io/DT/server.html
```

## **Extended study : PMM2-CDG**

Per each configuration, a plot of AUC (y) grouped by maximum logFC to simulate (x) will be plotted, grouping by number of genes (rows) and DEG proportion (columns) simulated:

## **Vote systems results : PMM2-CDG**

Measures (y) comparisson for all possible vote systems thresholds (x) grouping by number of replicates (columns) and each configuration (grid set):

##
