# Supplementary material for: Gene expression analysis method integration and co-expression module detection applied to rare glucide metabolism disorders using ExpHunterSuite
Source: Sci Rep. 2021 Jul 23;11:15062. doi: 10.1038/s41598-021-94343-w (PMC8302605; doi:10.1038/s41598-021-94343-w)
Supplement: Supplementary file 6 — Supplementary Report 2. [file 41598_2021_94343_MOESM6_ESM.html]

DEG detection method comparison for spikein dataset


# DEG detection method comparison for spikein dataset

#### James Perkins

# single

### Results per mix

#### Mix3\_vs\_Mix2

| Method | Comparison | TP | FP | TN | FN | Accuracy | Precision | Recall | Specificity | FMeasure | AUC |
| --- | --- | --- | --- | --- | --- | --- | --- | --- | --- | --- | --- |
| edgeR | Mix3\_vs\_Mix2 | 18 | 13 | 55303 | 1 | 1.000 | 0.581 | 0.947 | 1 | 0.720 | 0.974 |
| DESeq2 | Mix3\_vs\_Mix2 | 18 | 15 | 55301 | 1 | 1.000 | 0.545 | 0.947 | 1 | 0.692 | 0.993 |
| limma | Mix3\_vs\_Mix2 | 15 | 8 | 55308 | 4 | 1.000 | 0.652 | 0.789 | 1 | 0.714 | 0.993 |
| NOISeq | Mix3\_vs\_Mix2 | 18 | 27 | 55289 | 1 | 0.999 | 0.400 | 0.947 | 1 | 0.562 | 0.984 |
| combined | Mix3\_vs\_Mix2 | 18 | 14 | 55302 | 1 | 1.000 | 0.562 | 0.947 | 1 | 0.706 | 0.998 |

| Comparison | Cut | TP | FP | TN | FN | Accuracy | Precision | Recall | Specificity | FMeasure |
| --- | --- | --- | --- | --- | --- | --- | --- | --- | --- | --- |
| Mix3\_vs\_Mix2 | Cut\_1 | 18 | 28 | 55288 | 1 | 0.999 | 0.391 | 0.947 | 0.999 | 0.554 |
| Mix3\_vs\_Mix2 | Cut\_2 | 18 | 15 | 55301 | 1 | 1.000 | 0.545 | 0.947 | 1.000 | 0.692 |
| Mix3\_vs\_Mix2 | Cut\_3 | 18 | 13 | 55303 | 1 | 1.000 | 0.581 | 0.947 | 1.000 | 0.720 |
| Mix3\_vs\_Mix2 | Cut\_4 | 18 | 13 | 55303 | 1 | 1.000 | 0.581 | 0.947 | 1.000 | 0.720 |
| Mix3\_vs\_Mix2 | Combined | 18 | 14 | 55302 | 1 | 1.000 | 0.562 | 0.947 | 1.000 | 0.706 |
| Mix3\_vs\_Mix2 | Naive\_Bayes | 18 | 14 | 55302 | 1 | 1.000 | 0.562 | 0.947 | 1.000 | 0.706 |

#### Mix3\_vs\_Mix1

| Method | Comparison | TP | FP | TN | FN | Accuracy | Precision | Recall | Specificity | FMeasure | AUC |
| --- | --- | --- | --- | --- | --- | --- | --- | --- | --- | --- | --- |
| edgeR | Mix3\_vs\_Mix1 | 16 | 16 | 55300 | 3 | 1.000 | 0.500 | 0.842 | 1.000 | 0.627 | 0.921 |
| DESeq2 | Mix3\_vs\_Mix1 | 16 | 16 | 55300 | 3 | 1.000 | 0.500 | 0.842 | 1.000 | 0.627 | 0.953 |
| limma | Mix3\_vs\_Mix1 | 12 | 10 | 55306 | 7 | 1.000 | 0.545 | 0.632 | 1.000 | 0.585 | 0.953 |
| NOISeq | Mix3\_vs\_Mix1 | 16 | 41 | 55275 | 3 | 0.999 | 0.281 | 0.842 | 0.999 | 0.421 | 0.940 |
| combined | Mix3\_vs\_Mix1 | 16 | 16 | 55300 | 3 | 1.000 | 0.500 | 0.842 | 1.000 | 0.627 | 0.940 |

| Comparison | Cut | TP | FP | TN | FN | Accuracy | Precision | Recall | Specificity | FMeasure |
| --- | --- | --- | --- | --- | --- | --- | --- | --- | --- | --- |
| Mix3\_vs\_Mix1 | Cut\_1 | 16 | 41 | 55275 | 3 | 0.999 | 0.281 | 0.842 | 0.999 | 0.421 |
| Mix3\_vs\_Mix1 | Cut\_2 | 16 | 16 | 55300 | 3 | 1.000 | 0.500 | 0.842 | 1.000 | 0.627 |
| Mix3\_vs\_Mix1 | Cut\_3 | 16 | 16 | 55300 | 3 | 1.000 | 0.500 | 0.842 | 1.000 | 0.627 |
| Mix3\_vs\_Mix1 | Cut\_4 | 16 | 16 | 55300 | 3 | 1.000 | 0.500 | 0.842 | 1.000 | 0.627 |
| Mix3\_vs\_Mix1 | Combined | 16 | 16 | 55300 | 3 | 1.000 | 0.500 | 0.842 | 1.000 | 0.627 |
| Mix3\_vs\_Mix1 | Naive\_Bayes | 16 | 16 | 55300 | 3 | 1.000 | 0.500 | 0.842 | 1.000 | 0.627 |

#### Mix2\_vs\_Mix1

| Method | Comparison | TP | FP | TN | FN | Accuracy | Precision | Recall | Specificity | FMeasure | AUC |
| --- | --- | --- | --- | --- | --- | --- | --- | --- | --- | --- | --- |
| edgeR | Mix2\_vs\_Mix1 | 17 | 15 | 55301 | 2 | 1 | 0.531 | 0.895 | 1 | 0.667 | 0.974 |
| DESeq2 | Mix2\_vs\_Mix1 | 17 | 15 | 55301 | 2 | 1 | 0.531 | 0.895 | 1 | 0.667 | 0.993 |
| limma | Mix2\_vs\_Mix1 | 12 | 11 | 55305 | 7 | 1 | 0.522 | 0.632 | 1 | 0.571 | 0.986 |
| NOISeq | Mix2\_vs\_Mix1 | 17 | 16 | 55300 | 2 | 1 | 0.515 | 0.895 | 1 | 0.654 | 0.972 |
| combined | Mix2\_vs\_Mix1 | 17 | 15 | 55301 | 2 | 1 | 0.531 | 0.895 | 1 | 0.667 | 0.986 |

| Comparison | Cut | TP | FP | TN | FN | Accuracy | Precision | Recall | Specificity | FMeasure |
| --- | --- | --- | --- | --- | --- | --- | --- | --- | --- | --- |
| Mix2\_vs\_Mix1 | Cut\_1 | 17 | 16 | 55300 | 2 | 1 | 0.515 | 0.895 | 1 | 0.654 |
| Mix2\_vs\_Mix1 | Cut\_2 | 17 | 15 | 55301 | 2 | 1 | 0.531 | 0.895 | 1 | 0.667 |
| Mix2\_vs\_Mix1 | Cut\_3 | 17 | 15 | 55301 | 2 | 1 | 0.531 | 0.895 | 1 | 0.667 |
| Mix2\_vs\_Mix1 | Cut\_4 | 17 | 15 | 55301 | 2 | 1 | 0.531 | 0.895 | 1 | 0.667 |
| Mix2\_vs\_Mix1 | Combined | 17 | 15 | 55301 | 2 | 1 | 0.531 | 0.895 | 1 | 0.667 |
| Mix2\_vs\_Mix1 | Naive\_Bayes | 18 | 15 | 55301 | 1 | 1 | 0.545 | 0.947 | 1 | 0.692 |

### Comparing methods

#### AUC vals:

| Comparison | DESeq2 | NOISeq | combined | edgeR | limma |
| --- | --- | --- | --- | --- | --- |
| Mix2\_vs\_Mix1 | 0.993 | 0.972 | 0.986 | 0.974 | 0.986 |
| Mix3\_vs\_Mix1 | 0.953 | 0.940 | 0.940 | 0.921 | 0.953 |
| Mix3\_vs\_Mix2 | 0.993 | 0.984 | 0.998 | 0.974 | 0.993 |

#### F1 vals:

| Comparison | DESeq2 | NOISeq | combined | edgeR | limma |
| --- | --- | --- | --- | --- | --- |
| Mix2\_vs\_Mix1 | 0.667 | 0.654 | 0.667 | 0.667 | 0.571 |
| Mix3\_vs\_Mix1 | 0.627 | 0.421 | 0.627 | 0.627 | 0.585 |
| Mix3\_vs\_Mix2 | 0.692 | 0.562 | 0.706 | 0.720 | 0.714 |

|  | FP | FN | Precision | Recall | FMeasure | AUC |
| --- | --- | --- | --- | --- | --- | --- |
| combined | 15.00 | 2 | 0.531 | 0.895 | 0.667 | 0.974 |
| DESeq2 | 15.33 | 2 | 0.526 | 0.895 | 0.662 | 0.979 |
| edgeR | 14.67 | 2 | 0.537 | 0.895 | 0.671 | 0.956 |
| limma | 9.67 | 6 | 0.573 | 0.684 | 0.624 | 0.977 |
| NOISeq | 28.00 | 2 | 0.399 | 0.895 | 0.546 | 0.965 |

#### Plot AUC and F1

### Comparing vote cutoffs

#### Accuracy vals:

| Comparison | Combined | Cut\_1 | Cut\_2 | Cut\_3 | Cut\_4 | Naive\_Bayes |
| --- | --- | --- | --- | --- | --- | --- |
| Mix2\_vs\_Mix1 | 1 | 1.000 | 1 | 1 | 1 | 1 |
| Mix3\_vs\_Mix1 | 1 | 0.999 | 1 | 1 | 1 | 1 |
| Mix3\_vs\_Mix2 | 1 | 0.999 | 1 | 1 | 1 | 1 |

#### F1 vals:

| Comparison | Combined | Cut\_1 | Cut\_2 | Cut\_3 | Cut\_4 | Naive\_Bayes |
| --- | --- | --- | --- | --- | --- | --- |
| Mix2\_vs\_Mix1 | 0.667 | 0.654 | 0.667 | 0.667 | 0.667 | 0.692 |
| Mix3\_vs\_Mix1 | 0.627 | 0.421 | 0.627 | 0.627 | 0.627 | 0.627 |
| Mix3\_vs\_Mix2 | 0.706 | 0.554 | 0.692 | 0.720 | 0.720 | 0.706 |

|  | FP | FN | Precision | Recall | FMeasure |
| --- | --- | --- | --- | --- | --- |
| Combined | 15.0 | 2.00 | 0.531 | 0.895 | 0.667 |
| Cut\_1 | 28.3 | 2.00 | 0.396 | 0.895 | 0.543 |
| Cut\_2 | 15.3 | 2.00 | 0.526 | 0.895 | 0.662 |
| Cut\_3 | 14.7 | 2.00 | 0.537 | 0.895 | 0.671 |
| Cut\_4 | 14.7 | 2.00 | 0.537 | 0.895 | 0.671 |
| Naive\_Bayes | 15.0 | 1.67 | 0.536 | 0.912 | 0.675 |

#### Plot Accuracy and F1

# multi

### Results per mix

#### Mix3\_vs\_Mix2

| Method | Comparison | TP | FP | TN | FN | Accuracy | Precision | Recall | Specificity | FMeasure | AUC |
| --- | --- | --- | --- | --- | --- | --- | --- | --- | --- | --- | --- |
| edgeR | Mix3\_vs\_Mix2 | 8 | 23 | 55300 | 4 | 1.000 | 0.258 | 0.667 | 1.000 | 0.372 | 0.916 |
| DESeq2 | Mix3\_vs\_Mix2 | 10 | 23 | 55300 | 2 | 1.000 | 0.303 | 0.833 | 1.000 | 0.444 | 0.978 |
| limma | Mix3\_vs\_Mix2 | 5 | 18 | 55305 | 7 | 1.000 | 0.217 | 0.417 | 1.000 | 0.286 | 0.944 |
| NOISeq | Mix3\_vs\_Mix2 | 9 | 36 | 55287 | 3 | 0.999 | 0.200 | 0.750 | 0.999 | 0.316 | 0.898 |
| combined | Mix3\_vs\_Mix2 | 9 | 23 | 55300 | 3 | 1.000 | 0.281 | 0.750 | 1.000 | 0.409 | 0.963 |

| Comparison | Cut | TP | FP | TN | FN | Accuracy | Precision | Recall | Specificity | FMeasure |
| --- | --- | --- | --- | --- | --- | --- | --- | --- | --- | --- |
| Mix3\_vs\_Mix2 | Cut\_1 | 10 | 36 | 55287 | 2 | 0.999 | 0.217 | 0.833 | 0.999 | 0.345 |
| Mix3\_vs\_Mix2 | Cut\_2 | 10 | 23 | 55300 | 2 | 1.000 | 0.303 | 0.833 | 1.000 | 0.444 |
| Mix3\_vs\_Mix2 | Cut\_3 | 8 | 23 | 55300 | 4 | 1.000 | 0.258 | 0.667 | 1.000 | 0.372 |
| Mix3\_vs\_Mix2 | Cut\_4 | 8 | 23 | 55300 | 4 | 1.000 | 0.258 | 0.667 | 1.000 | 0.372 |
| Mix3\_vs\_Mix2 | Combined | 9 | 23 | 55300 | 3 | 1.000 | 0.281 | 0.750 | 1.000 | 0.409 |
| Mix3\_vs\_Mix2 | Naive\_Bayes | 9 | 23 | 55300 | 3 | 1.000 | 0.281 | 0.750 | 1.000 | 0.409 |

#### Mix3\_vs\_Mix1

| Method | Comparison | TP | FP | TN | FN | Accuracy | Precision | Recall | Specificity | FMeasure | AUC |
| --- | --- | --- | --- | --- | --- | --- | --- | --- | --- | --- | --- |
| edgeR | Mix3\_vs\_Mix1 | 12 | 20 | 55303 | 0 | 1.000 | 0.375 | 1.000 | 1.000 | 0.545 | 1.000 |
| DESeq2 | Mix3\_vs\_Mix1 | 12 | 20 | 55303 | 0 | 1.000 | 0.375 | 1.000 | 1.000 | 0.545 | 1.000 |
| limma | Mix3\_vs\_Mix1 | 7 | 15 | 55308 | 5 | 1.000 | 0.318 | 0.583 | 1.000 | 0.412 | 0.989 |
| NOISeq | Mix3\_vs\_Mix1 | 12 | 45 | 55278 | 0 | 0.999 | 0.211 | 1.000 | 0.999 | 0.348 | 0.943 |
| combined | Mix3\_vs\_Mix1 | 12 | 20 | 55303 | 0 | 1.000 | 0.375 | 1.000 | 1.000 | 0.545 | 1.000 |

| Comparison | Cut | TP | FP | TN | FN | Accuracy | Precision | Recall | Specificity | FMeasure |
| --- | --- | --- | --- | --- | --- | --- | --- | --- | --- | --- |
| Mix3\_vs\_Mix1 | Cut\_1 | 12 | 45 | 55278 | 0 | 0.999 | 0.211 | 1 | 0.999 | 0.348 |
| Mix3\_vs\_Mix1 | Cut\_2 | 12 | 20 | 55303 | 0 | 1.000 | 0.375 | 1 | 1.000 | 0.545 |
| Mix3\_vs\_Mix1 | Cut\_3 | 12 | 20 | 55303 | 0 | 1.000 | 0.375 | 1 | 1.000 | 0.545 |
| Mix3\_vs\_Mix1 | Cut\_4 | 12 | 20 | 55303 | 0 | 1.000 | 0.375 | 1 | 1.000 | 0.545 |
| Mix3\_vs\_Mix1 | Combined | 12 | 20 | 55303 | 0 | 1.000 | 0.375 | 1 | 1.000 | 0.545 |
| Mix3\_vs\_Mix1 | Naive\_Bayes | 12 | 20 | 55303 | 0 | 1.000 | 0.375 | 1 | 1.000 | 0.545 |

#### Mix2\_vs\_Mix1

| Method | Comparison | TP | FP | TN | FN | Accuracy | Precision | Recall | Specificity | FMeasure | AUC |
| --- | --- | --- | --- | --- | --- | --- | --- | --- | --- | --- | --- |
| edgeR | Mix2\_vs\_Mix1 | 11 | 21 | 55302 | 1 | 1 | 0.344 | 0.917 | 1 | 0.500 | 0.958 |
| DESeq2 | Mix2\_vs\_Mix1 | 11 | 21 | 55302 | 1 | 1 | 0.344 | 0.917 | 1 | 0.500 | 0.989 |
| limma | Mix2\_vs\_Mix1 | 10 | 13 | 55310 | 2 | 1 | 0.435 | 0.833 | 1 | 0.571 | 0.989 |
| NOISeq | Mix2\_vs\_Mix1 | 11 | 22 | 55301 | 1 | 1 | 0.333 | 0.917 | 1 | 0.489 | 0.978 |
| combined | Mix2\_vs\_Mix1 | 11 | 21 | 55302 | 1 | 1 | 0.344 | 0.917 | 1 | 0.500 | 0.978 |

| Comparison | Cut | TP | FP | TN | FN | Accuracy | Precision | Recall | Specificity | FMeasure |
| --- | --- | --- | --- | --- | --- | --- | --- | --- | --- | --- |
| Mix2\_vs\_Mix1 | Cut\_1 | 11 | 22 | 55301 | 1 | 1 | 0.333 | 0.917 | 1 | 0.489 |
| Mix2\_vs\_Mix1 | Cut\_2 | 11 | 21 | 55302 | 1 | 1 | 0.344 | 0.917 | 1 | 0.500 |
| Mix2\_vs\_Mix1 | Cut\_3 | 11 | 21 | 55302 | 1 | 1 | 0.344 | 0.917 | 1 | 0.500 |
| Mix2\_vs\_Mix1 | Cut\_4 | 11 | 21 | 55302 | 1 | 1 | 0.344 | 0.917 | 1 | 0.500 |
| Mix2\_vs\_Mix1 | Combined | 11 | 21 | 55302 | 1 | 1 | 0.344 | 0.917 | 1 | 0.500 |
| Mix2\_vs\_Mix1 | Naive\_Bayes | 11 | 22 | 55301 | 1 | 1 | 0.333 | 0.917 | 1 | 0.489 |

### Comparing methods

#### AUC vals:

| Comparison | DESeq2 | NOISeq | combined | edgeR | limma |
| --- | --- | --- | --- | --- | --- |
| Mix2\_vs\_Mix1 | 0.989 | 0.978 | 0.978 | 0.958 | 0.989 |
| Mix3\_vs\_Mix1 | 1.000 | 0.943 | 1.000 | 1.000 | 0.989 |
| Mix3\_vs\_Mix2 | 0.978 | 0.898 | 0.963 | 0.916 | 0.944 |

#### F1 vals:

| Comparison | DESeq2 | NOISeq | combined | edgeR | limma |
| --- | --- | --- | --- | --- | --- |
| Mix2\_vs\_Mix1 | 0.500 | 0.489 | 0.500 | 0.500 | 0.571 |
| Mix3\_vs\_Mix1 | 0.545 | 0.348 | 0.545 | 0.545 | 0.412 |
| Mix3\_vs\_Mix2 | 0.444 | 0.316 | 0.409 | 0.372 | 0.286 |

|  | FP | FN | Precision | Recall | FMeasure | AUC |
| --- | --- | --- | --- | --- | --- | --- |
| combined | 21.3 | 1.33 | 0.333 | 0.889 | 0.485 | 0.980 |
| DESeq2 | 21.3 | 1.00 | 0.341 | 0.917 | 0.497 | 0.989 |
| edgeR | 21.3 | 1.67 | 0.326 | 0.861 | 0.473 | 0.958 |
| limma | 15.3 | 4.67 | 0.323 | 0.611 | 0.423 | 0.974 |
| NOISeq | 34.3 | 1.33 | 0.248 | 0.889 | 0.384 | 0.939 |

#### Plot AUC and F1

### Comparing vote cutoffs

#### Accuracy vals:

| Comparison | Combined | Cut\_1 | Cut\_2 | Cut\_3 | Cut\_4 | Naive\_Bayes |
| --- | --- | --- | --- | --- | --- | --- |
| Mix2\_vs\_Mix1 | 1 | 1.000 | 1 | 1 | 1 | 1 |
| Mix3\_vs\_Mix1 | 1 | 0.999 | 1 | 1 | 1 | 1 |
| Mix3\_vs\_Mix2 | 1 | 0.999 | 1 | 1 | 1 | 1 |

#### F1 vals:

| Comparison | Combined | Cut\_1 | Cut\_2 | Cut\_3 | Cut\_4 | Naive\_Bayes |
| --- | --- | --- | --- | --- | --- | --- |
| Mix2\_vs\_Mix1 | 0.500 | 0.489 | 0.500 | 0.500 | 0.500 | 0.489 |
| Mix3\_vs\_Mix1 | 0.545 | 0.348 | 0.545 | 0.545 | 0.545 | 0.545 |
| Mix3\_vs\_Mix2 | 0.409 | 0.345 | 0.444 | 0.372 | 0.372 | 0.409 |

|  | FP | FN | Precision | Recall | FMeasure |
| --- | --- | --- | --- | --- | --- |
| Combined | 21.3 | 1.33 | 0.333 | 0.889 | 0.485 |
| Cut\_1 | 34.3 | 1.00 | 0.254 | 0.917 | 0.394 |
| Cut\_2 | 21.3 | 1.00 | 0.341 | 0.917 | 0.497 |
| Cut\_3 | 21.3 | 1.67 | 0.326 | 0.861 | 0.473 |
| Cut\_4 | 21.3 | 1.67 | 0.326 | 0.861 | 0.473 |
| Naive\_Bayes | 21.7 | 1.33 | 0.330 | 0.889 | 0.481 |

#### Plot Accuracy and F1

# all

### Results per mix

#### Mix3\_vs\_Mix2

| Method | Comparison | TP | FP | TN | FN | Accuracy | Precision | Recall | Specificity | FMeasure | AUC |
| --- | --- | --- | --- | --- | --- | --- | --- | --- | --- | --- | --- |
| edgeR | Mix3\_vs\_Mix2 | 26 | 5 | 55299 | 5 | 1 | 0.839 | 0.839 | 1 | 0.839 | 0.952 |
| DESeq2 | Mix3\_vs\_Mix2 | 28 | 5 | 55299 | 3 | 1 | 0.848 | 0.903 | 1 | 0.875 | 0.987 |
| limma | Mix3\_vs\_Mix2 | 20 | 3 | 55301 | 11 | 1 | 0.870 | 0.645 | 1 | 0.741 | 0.974 |
| NOISeq | Mix3\_vs\_Mix2 | 27 | 18 | 55286 | 4 | 1 | 0.600 | 0.871 | 1 | 0.711 | 0.951 |
| combined | Mix3\_vs\_Mix2 | 27 | 5 | 55299 | 4 | 1 | 0.844 | 0.871 | 1 | 0.857 | 0.984 |

| Comparison | Cut | TP | FP | TN | FN | Accuracy | Precision | Recall | Specificity | FMeasure |
| --- | --- | --- | --- | --- | --- | --- | --- | --- | --- | --- |
| Mix3\_vs\_Mix2 | Cut\_1 | 28 | 18 | 55286 | 3 | 1 | 0.609 | 0.903 | 1 | 0.727 |
| Mix3\_vs\_Mix2 | Cut\_2 | 28 | 5 | 55299 | 3 | 1 | 0.848 | 0.903 | 1 | 0.875 |
| Mix3\_vs\_Mix2 | Cut\_3 | 26 | 5 | 55299 | 5 | 1 | 0.839 | 0.839 | 1 | 0.839 |
| Mix3\_vs\_Mix2 | Cut\_4 | 26 | 5 | 55299 | 5 | 1 | 0.839 | 0.839 | 1 | 0.839 |
| Mix3\_vs\_Mix2 | Combined | 27 | 5 | 55299 | 4 | 1 | 0.844 | 0.871 | 1 | 0.857 |
| Mix3\_vs\_Mix2 | Naive\_Bayes | 27 | 5 | 55299 | 4 | 1 | 0.844 | 0.871 | 1 | 0.857 |

#### Mix3\_vs\_Mix1

| Method | Comparison | TP | FP | TN | FN | Accuracy | Precision | Recall | Specificity | FMeasure | AUC |
| --- | --- | --- | --- | --- | --- | --- | --- | --- | --- | --- | --- |
| edgeR | Mix3\_vs\_Mix1 | 28 | 4 | 55300 | 3 | 1.000 | 0.875 | 0.903 | 1.000 | 0.889 | 0.952 |
| DESeq2 | Mix3\_vs\_Mix1 | 28 | 4 | 55300 | 3 | 1.000 | 0.875 | 0.903 | 1.000 | 0.889 | 0.971 |
| limma | Mix3\_vs\_Mix1 | 19 | 3 | 55301 | 12 | 1.000 | 0.864 | 0.613 | 1.000 | 0.717 | 0.967 |
| NOISeq | Mix3\_vs\_Mix1 | 28 | 29 | 55275 | 3 | 0.999 | 0.491 | 0.903 | 0.999 | 0.636 | 0.941 |
| combined | Mix3\_vs\_Mix1 | 28 | 4 | 55300 | 3 | 1.000 | 0.875 | 0.903 | 1.000 | 0.889 | 0.963 |

| Comparison | Cut | TP | FP | TN | FN | Accuracy | Precision | Recall | Specificity | FMeasure |
| --- | --- | --- | --- | --- | --- | --- | --- | --- | --- | --- |
| Mix3\_vs\_Mix1 | Cut\_1 | 28 | 29 | 55275 | 3 | 0.999 | 0.491 | 0.903 | 0.999 | 0.636 |
| Mix3\_vs\_Mix1 | Cut\_2 | 28 | 4 | 55300 | 3 | 1.000 | 0.875 | 0.903 | 1.000 | 0.889 |
| Mix3\_vs\_Mix1 | Cut\_3 | 28 | 4 | 55300 | 3 | 1.000 | 0.875 | 0.903 | 1.000 | 0.889 |
| Mix3\_vs\_Mix1 | Cut\_4 | 28 | 4 | 55300 | 3 | 1.000 | 0.875 | 0.903 | 1.000 | 0.889 |
| Mix3\_vs\_Mix1 | Combined | 28 | 4 | 55300 | 3 | 1.000 | 0.875 | 0.903 | 1.000 | 0.889 |
| Mix3\_vs\_Mix1 | Naive\_Bayes | 28 | 4 | 55300 | 3 | 1.000 | 0.875 | 0.903 | 1.000 | 0.889 |

#### Mix2\_vs\_Mix1

| Method | Comparison | TP | FP | TN | FN | Accuracy | Precision | Recall | Specificity | FMeasure | AUC |
| --- | --- | --- | --- | --- | --- | --- | --- | --- | --- | --- | --- |
| edgeR | Mix2\_vs\_Mix1 | 28 | 4 | 55300 | 3 | 1 | 0.875 | 0.903 | 1 | 0.889 | 0.968 |
| DESeq2 | Mix2\_vs\_Mix1 | 28 | 4 | 55300 | 3 | 1 | 0.875 | 0.903 | 1 | 0.889 | 0.991 |
| limma | Mix2\_vs\_Mix1 | 22 | 1 | 55303 | 9 | 1 | 0.957 | 0.710 | 1 | 0.815 | 0.987 |
| NOISeq | Mix2\_vs\_Mix1 | 28 | 5 | 55299 | 3 | 1 | 0.848 | 0.903 | 1 | 0.875 | 0.975 |
| combined | Mix2\_vs\_Mix1 | 28 | 4 | 55300 | 3 | 1 | 0.875 | 0.903 | 1 | 0.889 | 0.983 |

| Comparison | Cut | TP | FP | TN | FN | Accuracy | Precision | Recall | Specificity | FMeasure |
| --- | --- | --- | --- | --- | --- | --- | --- | --- | --- | --- |
| Mix2\_vs\_Mix1 | Cut\_1 | 28 | 5 | 55299 | 3 | 1 | 0.848 | 0.903 | 1 | 0.875 |
| Mix2\_vs\_Mix1 | Cut\_2 | 28 | 4 | 55300 | 3 | 1 | 0.875 | 0.903 | 1 | 0.889 |
| Mix2\_vs\_Mix1 | Cut\_3 | 28 | 4 | 55300 | 3 | 1 | 0.875 | 0.903 | 1 | 0.889 |
| Mix2\_vs\_Mix1 | Cut\_4 | 28 | 4 | 55300 | 3 | 1 | 0.875 | 0.903 | 1 | 0.889 |
| Mix2\_vs\_Mix1 | Combined | 28 | 4 | 55300 | 3 | 1 | 0.875 | 0.903 | 1 | 0.889 |
| Mix2\_vs\_Mix1 | Naive\_Bayes | 29 | 4 | 55300 | 2 | 1 | 0.879 | 0.935 | 1 | 0.906 |

### Comparing methods

#### AUC vals:

| Comparison | DESeq2 | NOISeq | combined | edgeR | limma |
| --- | --- | --- | --- | --- | --- |
| Mix2\_vs\_Mix1 | 0.991 | 0.975 | 0.983 | 0.968 | 0.987 |
| Mix3\_vs\_Mix1 | 0.971 | 0.941 | 0.963 | 0.952 | 0.967 |
| Mix3\_vs\_Mix2 | 0.987 | 0.951 | 0.984 | 0.952 | 0.974 |

#### F1 vals:

| Comparison | DESeq2 | NOISeq | combined | edgeR | limma |
| --- | --- | --- | --- | --- | --- |
| Mix2\_vs\_Mix1 | 0.889 | 0.875 | 0.889 | 0.889 | 0.815 |
| Mix3\_vs\_Mix1 | 0.889 | 0.636 | 0.889 | 0.889 | 0.717 |
| Mix3\_vs\_Mix2 | 0.875 | 0.711 | 0.857 | 0.839 | 0.741 |

|  | FP | FN | Precision | Recall | FMeasure | AUC |
| --- | --- | --- | --- | --- | --- | --- |
| combined | 4.33 | 3.33 | 0.865 | 0.892 | 0.878 | 0.977 |
| DESeq2 | 4.33 | 3.00 | 0.866 | 0.903 | 0.884 | 0.983 |
| edgeR | 4.33 | 3.67 | 0.863 | 0.882 | 0.872 | 0.957 |
| limma | 2.33 | 10.67 | 0.897 | 0.656 | 0.758 | 0.976 |
| NOISeq | 17.33 | 3.33 | 0.647 | 0.892 | 0.741 | 0.955 |

#### Plot AUC and F1

### Comparing vote cutoffs

#### Accuracy vals:

| Comparison | Combined | Cut\_1 | Cut\_2 | Cut\_3 | Cut\_4 | Naive\_Bayes |
| --- | --- | --- | --- | --- | --- | --- |
| Mix2\_vs\_Mix1 | 1 | 1.000 | 1 | 1 | 1 | 1 |
| Mix3\_vs\_Mix1 | 1 | 0.999 | 1 | 1 | 1 | 1 |
| Mix3\_vs\_Mix2 | 1 | 1.000 | 1 | 1 | 1 | 1 |

#### F1 vals:

| Comparison | Combined | Cut\_1 | Cut\_2 | Cut\_3 | Cut\_4 | Naive\_Bayes |
| --- | --- | --- | --- | --- | --- | --- |
| Mix2\_vs\_Mix1 | 0.889 | 0.875 | 0.889 | 0.889 | 0.889 | 0.906 |
| Mix3\_vs\_Mix1 | 0.889 | 0.636 | 0.889 | 0.889 | 0.889 | 0.889 |
| Mix3\_vs\_Mix2 | 0.857 | 0.727 | 0.875 | 0.839 | 0.839 | 0.857 |

|  | FP | FN | Precision | Recall | FMeasure |
| --- | --- | --- | --- | --- | --- |
| Combined | 4.33 | 3.33 | 0.865 | 0.892 | 0.878 |
| Cut\_1 | 17.33 | 3.00 | 0.649 | 0.903 | 0.746 |
| Cut\_2 | 4.33 | 3.00 | 0.866 | 0.903 | 0.884 |
| Cut\_3 | 4.33 | 3.67 | 0.863 | 0.882 | 0.872 |
| Cut\_4 | 4.33 | 3.67 | 0.863 | 0.882 | 0.872 |
| Naive\_Bayes | 4.33 | 3.00 | 0.866 | 0.903 | 0.884 |

#### Plot Accuracy and F1
