# Supplementary material for: Gene expression analysis method integration and co-expression module detection applied to rare glucide metabolism disorders using ExpHunterSuite
Source: Sci Rep. 2021 Jul 23;11:15062. doi: 10.1038/s41598-021-94343-w (PMC8302605; doi:10.1038/s41598-021-94343-w)
Supplement: Supplementary file 7 — Supplementary Report 3. [file 41598_2021_94343_MOESM7_ESM.html]

FC Comparison for spikeins between DEG detection methods


# FC Comparison for spikeins between DEG detection methods

#### James Perkins

# single

### Mix2\_Mix1

### Mix3\_Mix1

### Mix4\_Mix1

### Mix3\_Mix2

### Mix4\_Mix2

### Mix4\_Mix3

# Correlation Matrix:

|  | Mix2\_Mix1 | Mix3\_Mix1 | Mix4\_Mix1 | Mix3\_Mix2 | Mix4\_Mix2 | Mix4\_Mix3 |
| --- | --- | --- | --- | --- | --- | --- |
| logFC\_DESeq2 | 0.9552497 | 0.9859323 | 0.8516294 | 0.9864248 | 0.8570169 | 0.8905170 |
| logFC\_edgeR | 0.9551911 | 0.9859569 | 0.8446026 | 0.9859190 | 0.8557915 | 0.8881744 |
| logFC\_limma | 0.9583709 | 0.9870396 | 0.8699591 | 0.9837751 | 0.8734158 | 0.9011874 |
| logFC\_NOISeq | 0.9553562 | 0.9860164 | 0.8753006 | 0.9851690 | 0.8773020 | 0.8968675 |
| mean\_logFCs | 0.9560748 | 0.9862857 | 0.8638451 | 0.9861029 | 0.8684504 | 0.8961399 |

# Average Correlation Values:

# multi

### Mix2\_Mix1

### Mix3\_Mix1

### Mix4\_Mix1

### Mix3\_Mix2

### Mix4\_Mix2

### Mix4\_Mix3

# Correlation Matrix:

|  | Mix2\_Mix1 | Mix3\_Mix1 | Mix4\_Mix1 | Mix3\_Mix2 | Mix4\_Mix2 | Mix4\_Mix3 |
| --- | --- | --- | --- | --- | --- | --- |
| logFC\_DESeq2 | 0.9800375 | 0.9816748 | 0.5469737 | 0.9727543 | 0.8266301 | 0.8125241 |
| logFC\_edgeR | 0.9800689 | 0.9817025 | 0.5763254 | 0.9727416 | 0.8349599 | 0.8166292 |
| logFC\_limma | 0.9820691 | 0.9821858 | 0.6653920 | 0.9736416 | 0.8951858 | 0.8074248 |
| logFC\_NOISeq | 0.9800260 | 0.9816446 | 0.6292535 | 0.9727391 | 0.8541163 | 0.8270021 |
| mean\_logFCs | 0.9805728 | 0.9818338 | 0.6060400 | 0.9730312 | 0.8543101 | 0.8168322 |

# Average Correlation Values:

# all

### Mix2\_Mix1

### Mix3\_Mix1

### Mix4\_Mix1

### Mix3\_Mix2

### Mix4\_Mix2

### Mix4\_Mix3

# Correlation Matrix:

|  | Mix2\_Mix1 | Mix3\_Mix1 | Mix4\_Mix1 | Mix3\_Mix2 | Mix4\_Mix2 | Mix4\_Mix3 |
| --- | --- | --- | --- | --- | --- | --- |
| logFC\_DESeq2 | 0.9640530 | 0.9823109 | 0.7825565 | 0.9813491 | 0.8440948 | 0.8822915 |
| logFC\_edgeR | 0.9640356 | 0.9823541 | 0.7871872 | 0.9811229 | 0.8477369 | 0.8828895 |
| logFC\_limma | 0.9667345 | 0.9825188 | 0.8292201 | 0.9791382 | 0.8714605 | 0.8903645 |
| logFC\_NOISeq | 0.9641085 | 0.9823732 | 0.8216068 | 0.9801979 | 0.8683875 | 0.8908949 |
| mean\_logFCs | 0.9647614 | 0.9824400 | 0.8084127 | 0.9810368 | 0.8599918 | 0.8881301 |

# Average Correlation Values:
